# The response of dominant and rare taxa for fungal diversity within different root environments to the cultivation of *Bt* and conventional cotton varieties

**DOI:** 10.1186/s40168-018-0570-9

**Published:** 2018-10-18

**Authors:** Peng Li, Yong Xue, Jialiang Shi, Aihu Pan, Xueming Tang, Feng Ming

**Affiliations:** 1 Biotechnology Research Institute, Shanghai Academy of Agricultural Sciences, Shanghai, 201106 China; 2 State Key Laboratory of Genetic Engineering, Institute of Genetics, Institute of Plant Biology, School of Life Sciences, Fudan University, Shanghai, 200433 China; 3 Institute of Eco-Environment and Plant Protection, Shanghai Academy of Agricultural Sciences, Shanghai, 201403 China; 4 Dezhou Academy of Agricultural Sciences, Dezhou, 253000 China

**Keywords:** Dominant taxa, Rare taxa, Illumina MiSeq sequencing, qPCR, *Bt* cotton, Fungal community composition

## Abstract

**Background:**

*Bacillus thuringiensis* (*Bt*) crops have been cultivated at a large scale over the past several decades, which have raised concern about unintended effects on natural environments. Microbial communities typically contain numerous rare taxa that make up the majority of community populations. However, the response of dominant and rare taxa for fungal diversity to the different root environments of *Bt* plants remains unclear.

**Results:**

We quantified fungal population sizes and community composition via quantitative PCR of ITS genes and 18S rRNA gene sequencing of, respectively, that were associated with *Bt* and conventional cotton variety rhizosphere soils from different plant growth stages. qPCR analyses indicated that fungal abundances reached their peak at the seedling stage and that the taproots and lateral root rhizospheres of the *Bt* cotton SGK321 were significantly different. However, no significant differences in population sizes were detected between the same root zones from *Bt* and the conventional cotton varieties. The overall patterns of fungal genera abundances followed that of the dominant genera, whereas overall patterns of fungal genera richness followed those of the rare genera. These results suggest that the dominant and rare taxa play different roles in the maintenance of rhizosphere microhabitat ecosystems. Cluster analyses indicated a separation of fungal communities based on the lateral roots or taproots from the three cotton varieties at the seedling stage, suggesting that root microhabitats had marked effects on fungal community composition. Redundancy analyses indicated that pH was more correlated to soil fungal community composition than Bt protein content.

**Conclusions:**

In conclusion, these results indicate that dominant and rare fungal taxa differentially contribute to community dynamics in different root microhabitats of both *Bt* and conventional cotton varieties. Moreover, these results showed that the rhizosphere fungal community of *Bt* cotton did not respond significantly to the presence of Bt protein when compared to the two conventional cotton varieties.

**Electronic supplementary material:**

The online version of this article (10.1186/s40168-018-0570-9) contains supplementary material, which is available to authorized users.

## Background

The *Bt* gene originating from *Bacillus thuringiensis* is widely used to confer pest resistance in plants [1]. Cultivation of these *Bacillus thuringiensis* (Bt) protein-containing crops has increased dramatically in recent years, with 75.4 million hectares of *Bt* crops planted in 2016, representing over 41% of all GM crops that were planted. Moreover, planting of *Bt* cotton globally accounted for 22.3 million hectares in 2016, and four countries each grew more than 1.0 million hectares [2]. The large-scale adoption of *Bt* crops can be attributed to the increased crop productivity and the reduced need for chemical pesticides, thereby resulting in a reduced environmental footprint for agriculture [3]. GM *Bt* crops produce insecticidal recombinant Cry1Ac protein, typically also in their root tissue. Such recombinant products may thus potentially enter the rhizosphere as an additional nutrient source for the soil microbial community. Consequently, one of the major potential environmental risks associated with *Bt* plants is their effect on soils and the potential to inhibit non-target organisms [4], such as fungi. Several studies have demonstrated that Bt proteins expressed by *Bt* crops enter soils through root exudates and then bind rapidly onto surface-active components, thereby becoming less accessible to microbial degradation but still retaining their insecticidal activity [5, 6]. Consequently, Bt proteins could accumulate in soils and may influence biological and chemical processes in addition to microbial community composition [7]. Fungi living in the rhizosphere can impact health, nutrition, and productivity of plants in agricultural ecosystems [8, 9], and understanding how fungal communities are affected by *Bt* crop cultivation is an essential aspect of elucidating soil biological processes at work in the rhizosphere by exposure to Bt proteins produced by *Bt* cotton. Li et al. [10] observed significant seasonal variation in the abundances and diversity of culturally viable fungi, but no significant differences could be attributable to the long-term cultivation of *Bt* cotton in the abundances of fungal populations or diversity. However, the communities identified appeared to be less taxonomically rich, and changes in the relative abundance of species were easy to overlook based on plate counts of cultivable organisms [11].

Natural microbial communities typically contain only a few dominant species, but a very large number of less abundant, rare taxa [12]. These low-abundance organisms can be representatives of novel microbial lineages [13, 14] and play crucial roles in biogeochemical cycles and metabolic fluxes [15, 16]. Rare taxa, in particular, have been reported to play important roles in the rhizosphere communities of *Bt* and conventional maize varieties [4]. For example, Hua et al. [17] found that dominant and rare taxa exhibited different ecological roles in acid mine drainage communities. Qi et al. [18] assessed fungal population dynamics in soils planted with *Bt* cotton and its conventional parental line using 18S and ITS rDNA gene FLX-pyrosequencing. However, little information is available regarding the response of dominant and rare fungal taxa to root exudates, including Bt protein. Studies on the importance of dominant and rare taxa for fungal diversity in the rhizosphere of GM crops expressing Bt proteins revealed by Illumina MiSeq sequencing contribute to their general environmental risk assessment, which is required to evaluate their safety and sustainable use in agriculture.

The root system plays a large role in the acquisition of resources in natural heterogeneous soil environments. Moreover, it has been proposed that root branching and root system architecture (RSA) play a significant role in determining the quantitative and qualitative composition of exudates, thereby modulating shifts in the diversity of soil microbial communities [19]. Yang and Crowley [20] observed that rhizosphere bacterial communities are substantially different in different root zones and that rhizosphere communities may be altered by changes in the root exudate composition. The RSA of cotton is typically composed of a primary (tap) root and lateral roots. A few studies have investigated the effects of consecutive cultivation of *Bt* cotton on soil microbe-mediated enzymatic properties and microbial biomass [21, 22]. However, no studies have investigated these effects on different root zones nor the effects of Cry proteins released in *Bt* cotton root exudates on fungal community structure in rhizospheres. Hence, investigating the effects of microhabitats within different root zones would be valuable to assess and precisely monitor the impacts of genetically modified plants on agro-ecosystems, and especially for dicotyledonous species.

The aim of this study was to characterize the dynamics of rhizosphere fungal community composition associated with *Bt* cotton SGK321 at different growth stages. Specifically, we assessed the response of dominant and rare fungal taxa to *Bt* cotton cultivation within different root environments (taproots and lateral roots) using qPCR of ITS gene abundances and high-resolution sequencing of 18S rRNA genes using Illumina MiSeq sequencing. To appropriately contextualize the potential differences caused by genetic modification, controls included two other cotton varieties that were cultivated in the same field and subjected to the same analyses as the lateral roots and taproots of *Bt* cotton. A specific evaluation was conducted of the possible links between soil Bt protein content and shifts in fungal community composition.

## Methods

### Experimental design and soil sampling

The transgenic *Bt* cotton variety SGK321 that produces Bt protein to inhibit bollworms was developed by the Shijiazhuang Academy of Agricultural and Forestry Sciences (China) and approved for commercial use by the Chinese government in 2002. A conventional parental cotton line of SY321 was used for comparison, in addition to one conventional variety XLZ13 that is widely cultivated. Seeds of SGK321 and SY321 were obtained from the Institute of Cotton Research of CAAS (Anyang, China), and those of XLZ13 from Economical Crop Research Institute of Xinjiang Academy of Agricultural Sciences (Urumqi, China). Plants of the three cotton varieties were grown from seeds in adjoining fields under the same environmental conditions and field management strategies. Each field comprised an area of 30 m × 30 m, with three replicates of each treatment arranged in a randomized block experimental design. Ten meters at both ends of each field was not used to avoid marginal effects. The survey was conducted in fields of the Dezhou Academy of Agricultural Sciences (Shandong, China). The field study did not involve endangered or protected species, and the specific location of this study was 37° 21′ N, 116° 20′ E. The fore-crop of the experimental field was a conventional wheat variety, and the wheat straw and litter were cleaned out before planting the cotton plants. The soil was classified as a sandy loam soil with 11.23 ± 0.89 g/kg organic matter, 1.72 ± 0.08 g/kg total nitrogen (N), 68.24 ± 5.78 mg/kg alk-hydr N, 17.54 ± 1.76 mg/kg available P, 109.78 ± 8.78 mg/kg available K, and a pH of 7.22 ± 0.35 (soil to water ratio of 1:2.5). The field experiment was conducted on March 30, 2016, according to the conventional local agriculture operations. In contrast to the two control varieties SY321 or XLZ13, SGK321showed a higher resistance against the cotton worm, which can especially prevent the normal growth and development of cotton worm. Moreover, the lint yield of SGK321 before frost was found to be higher than that of SY321 (or XLZ13), while its growth period is shorter than that of the two control varieties. The taproots and lateral root rhizospheres and bulk soils were sampled four times, corresponding to the major growth stages of cotton: seedling (July), budding (August), flowering (September), and bolling (October). After weeds and litter were removed from the soil surface, two types of soil samples were harvested. The bulk soil was excised from at least 4 mm away from any roots, and soils within 2 mm of the root surface were considered rhizosphere soils [23]. Plants were gently removed from the soils, and rhizospheres were collected by gently shaking the roots to dislodge small adhering soil clumps [24]. Rhizosphere samples were collected from five randomly selected plants in each plot and mixed to form a composite sample. Sampling was conducted in triplicate on each sampling day. The samples were placed on ice in a cooler and transported on the same day to the laboratory, where they were passed through a 2.0-mm sieve. After sieving, a portion of the soil sample was stored at − 80 °C for DNA extraction, and the remaining portion was stored at 4 °C for soil property and Bt protein content analysis.

### Analysis of soil physicochemical properties and soil microbial DNA extraction

To assess Bt protein content, up to 0.5 g of soil was added to a 2-mL microcentrifuge tube. One milliliter of extraction buffer (0.1 M Na_2_CO_3_, 0.1 M NaHCO_3_, 5.0 mM EDTA, 50 mM Na_4_P_2_O_7_.10H_2_O, 0.1%Triton X-100, pH 10) was then added to fill the tube, and the sample was mixed for 30 min at room temperature, followed by centrifugation for 5 min at 4 °C at 12,000*g*. The supernatant was collected, and Bt protein content was measured using a commercial ELISA kit (QuantiPlate™ Kit for Cry1Ab/Cry1Ac, EnviroLogix Inc., Portland, Maine, USA) according to the manufacturer’s protocol. Soil pH was determined using a pH meter (FE20-FiveEasy™ pH, Mettler Toledo, Germany) at a ratio of 1:2.5 (weight/volume) soil to distilled water. Soil microbial DNA was extracted as previously described by Li et al. [25].

### Quantification of fungal communities

We conducted quantitative PCR to determine population sizes of the rhizosphere and bulk soil fungal communities using the universal fungal ITS gene primers NSI1 and 58A2R [26]. Standard curves were generated using tenfold serial dilutions of a plasmid containing the ITS gene of *Aspergillus* sp. isolated from detritusphere. Each PCR reaction contained 5 μl × 2 SYBR qPCR Premix Ex Taq™ (TaKaRa Biotechnology (Dalian) Co., Ltd.), 0.25 μl each of 10 mM forward and reverse primers, 0.5 μl of deionized distilled water, and 4 μl of either standard or extracted soil DNA. PCR was performed on a StepOnePlus™ Real-Time PCR System (Applied Biosystems, Foster City, CA, USA) using a program, including initial denaturation at 95 °C for 3 min, followed by 40 cycles at 94 °C for 30 s, 53 °C for 30 s, and 72 °C for 45 s, with a final extension at 72 °C for 5 min. The copy number of fungal ITS rRNA genes was calculated using a regression equation to convert the cycle threshold (Ct) value to a known number of copies in the standards. All of the qPCR reactions were run in triplicate for each soil sample. The average fungal PCR efficiency was 93.3% with an *R*^2^ of the standard curves of 0.998.

### Fungal 18S rRNA gene Illumina MiSeq sequencing and sequence processing

A total of 72 independent samples that were obtained from the taproot and lateral root rhizospheres from the three cotton varieties (SGK321, SY321, and XLZ13) were selected for Illumina MiSeq sequencing of 18S rRNA genes (Additional file 1: Table S1). Fungal 18S rRNA genes were amplified using the primers SSU0817F (5′- TTA GCA TGG AAT AAT RRA ATA GGA -3′) and SSU1196R (5′- TCT GGA CCT GGT GAG TTT CC -3′) on a PCR thermal cycler system (GeneAmp 9700, ABI, USA). PCR reactions were conducted using the following program: 3 min of denaturation at 95 °C, followed by 35 cycles of 95 °C for 30 s; annealing at 53 °C for 30 s; and elongation at 72 °C for 45 s, followed by a final extension at 72 °C for 10 min. PCR reactions were performed in triplicate in 20-μl reaction mixtures containing 4 μl of × 5 FastPfu buffer, 2 μl of 2.5 mM dNTPs, 0.8 μl of each primer (5 μM), 0.2 μl of BSA, 0.4 μl of FastPfu polymerase, 10 ng of template DNA, and deionized distilled water for a total volume of 20 μl. The resultant PCR products were extracted from a 2% agarose gel and further purified using an AxyPrep DNA Gel Extraction Kit (Axygen Biosciences, Union City, CA, USA), followed by quantification using a QuantiFluor™-ST (Promega, USA) system according to the manufacturer’s protocols. Purified amplicons were pooled in equimolar concentrations and paired-end sequenced (2 × 300 bp) on the Illumina MiSeq platform (Illumina, San Diego, USA) according to the standard protocols at Majorbio Bio-Pharm Technology Co., Ltd. (Shanghai, China). A total of 12 independent bulk soil samples which were included in this study as the control group were also selected for Illumina MiSeq sequencing. All of the fungal 18S rRNA gene sequence data of rhizosphere and bulk soil analyzed in this study have been deposited into the NCBI Sequence Read Archive (SRA) database under the accession numbers SRP126911 and SRP154528, respectively.

Raw FASTQ files were de-multiplexed, quality filtered, and analyzed with QIIME Pipeline Version 1.7.0 (http://qiime.org/tutorials/tutorial.html) [27], and the paired reads were joined with fast length adjustment of short reads (FLASH) software [28] using the following criteria: (i) reads were truncated at any region receiving an average quality score < 20 over a 50-bp sliding window, (ii) reads containing > 2 nucleotide mismatches to primers were discarded along with reads containing ambiguous bases, and (iii) sequences with > 10 bp overlap were merged according to their overlapping sequence. Quality-filtered reads were then clustered into operational taxonomic units (OTUs) at a 97% similarity cutoff using the UPARSE algorithm (version 7.1 http://drive5.com/uparse/). Additionally, chimeric sequences were identified and removed using UCHIME [29]. The taxonomy of each 18S rRNA gene sequence was assessed using the RDP Classifier algorithm (http://rdp.cme.msu.edu) and the Silva (123/18) 18S rRNA database with a confidence threshold of 70%.

### Statistical analysis

Alpha diversity indices (Good’s coverage, Shannon diversity index, and Simpson diversity index) were calculated in QIIME (version 1.7.0) [27]. Statistically significant differences in soil pH, Bt protein content, fungal alpha diversity, ITS rRNA gene copies, and relative abundances of different fungal taxa among samples at each sampling time were examined using one-way analysis of variance (ANOVA) tests in SPSS 19.0 (SPSS Institute, Inc., 2010). Significant differences were considered as *P* < 0.05. Dominant, rare, and responsive fungal taxa at different taxonomic ranks were identified according to the methods described by Dohrmann et al. [4]. An analysis of similarities (ANOSIM) was performed using QIIME 1.8.0 software [27] to determine whether different group samples had significantly different microbial communities. Principal component analysis (PCA) based on Bray-Curtis similarities was performed using the R software package (https://www.r-project.org/). Redundancy analysis (RDA) was used to determine the environmental variables that most correlated to fungal community compositional differences, and the results were used to construct a soil property matrix for variation partitioning analysis in R v.2.8.1 using the “vegan” package (v.1.15-1) [30]. Cluster analysis of fungal communities was conducted based on Bray-Curtis dissimilarities using the “picante” and “vegan” packages in the R environment (R Development Core Team, 2006).

## Results

### Fungal population sizes in cotton rhizospheres and bulk soils

**Fig. 1:**
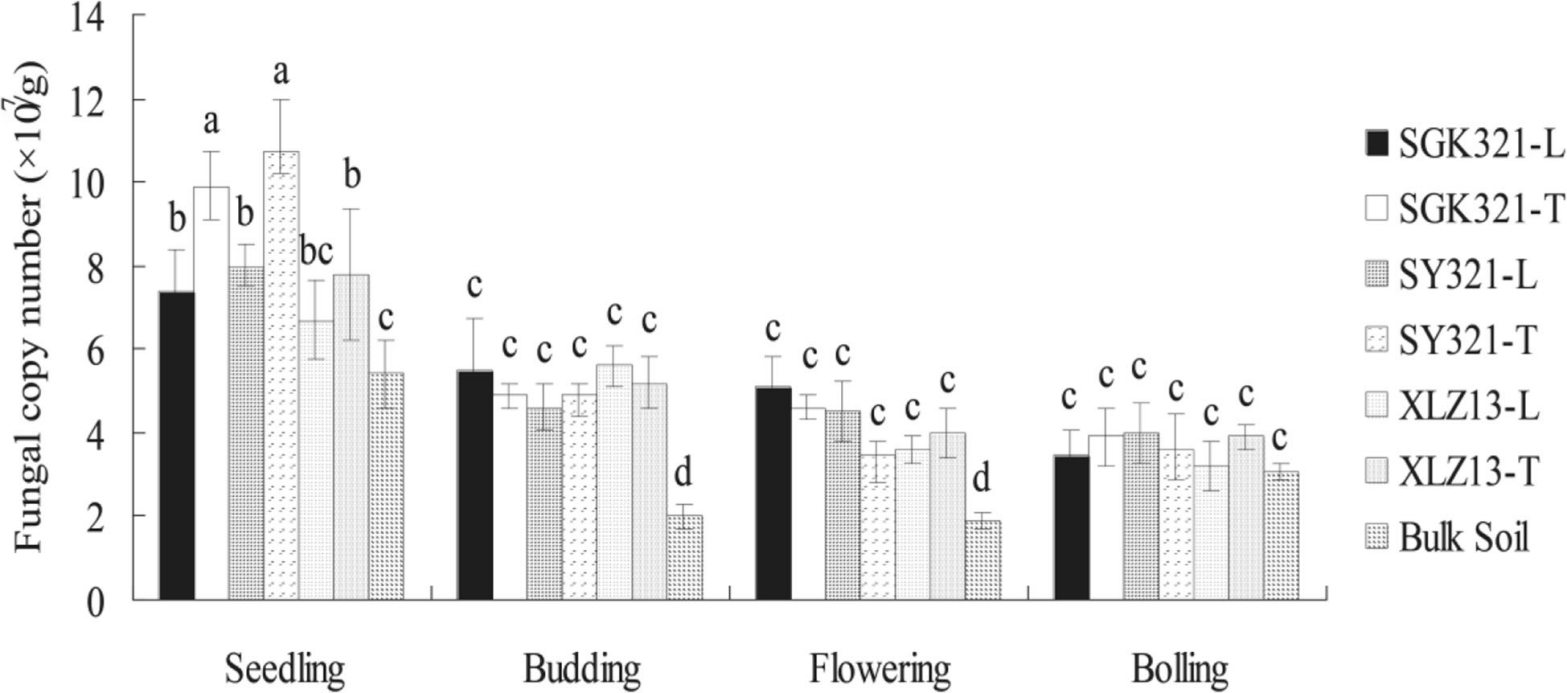
Fungal ITS rRNA gene copy numbers from the rhizosphere and bulk soils as determined by qPCR (mean ± SD)

The abundances of fungi in the 72 rhizosphere and 12 bulk soil samples ranged from 1.90 × 10^7^ to 10.71 × 10^7^ ITS rRNA gene copy numbers per gram dry soil (Fig. 1), with peak sizes at the seedling stage. Copy numbers in SGK321 and SY321 (or XLZ13) were not significantly different at the budding, flowering, and bolling stages. Notably, the population size of rhizosphere fungi associated with the taproot of SGK321 at the seedling stage was significantly higher compared to the lateral roots, suggesting that the root microhabitat was influential. There were no significant differences between fungal community sizes between rhizospheres of SGK321 and SY321 from the same root zones, suggesting that fungal abundance was not affected by the production of Bt proteins in the root tissue of SGK321. Moreover, the fungal population sizes of rhizosphere soil within the three cotton varieties were significantly higher than those of bulk soil at seedling, budding, and flowering stages, but no significant difference was noted between rhizosphere and bulk soils at bolling stage.

### Taxonomic assignment of the 18S rRNA gene sequences of rhizosphere fungi

**Fig. 2:**
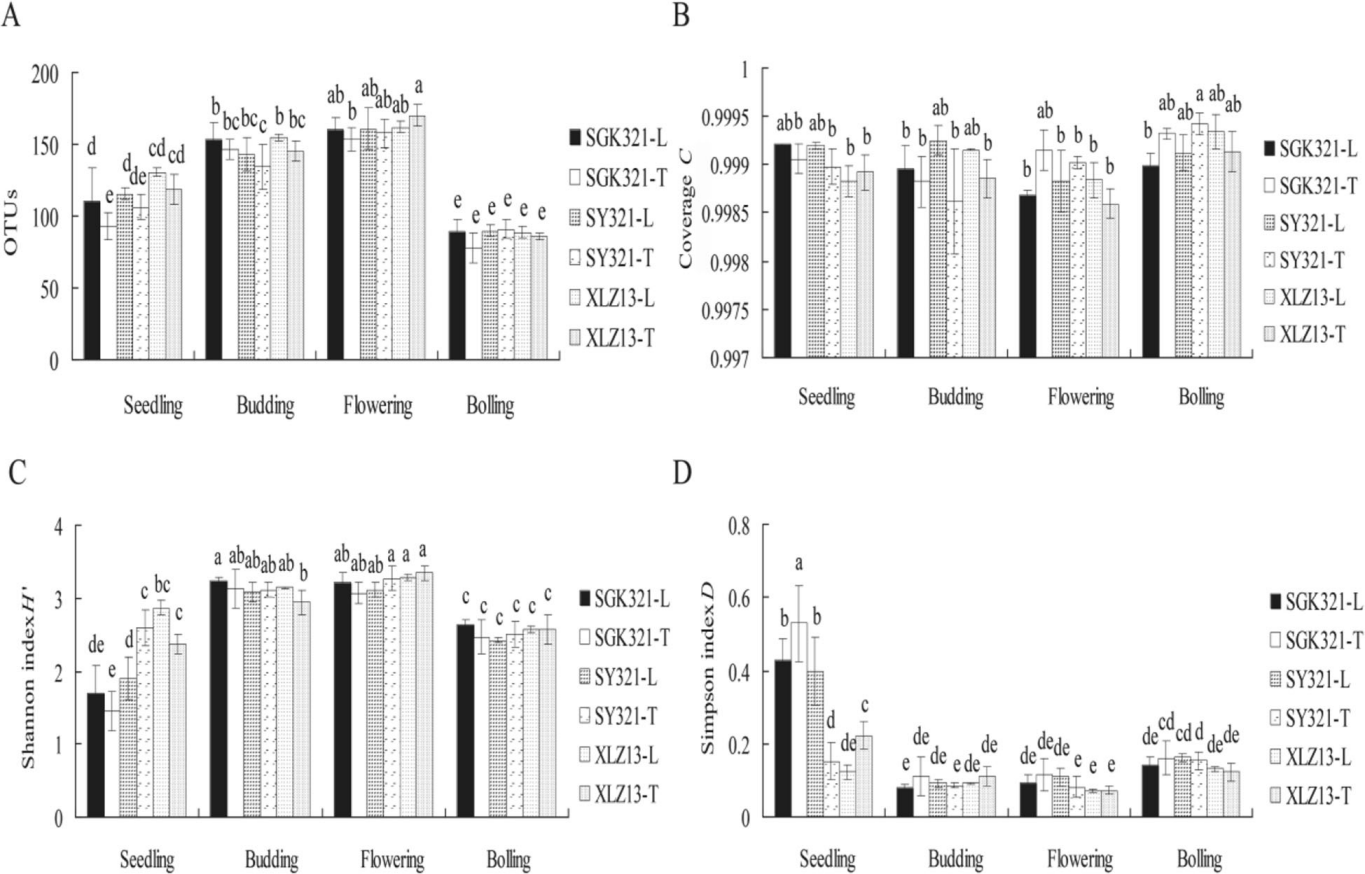
Number of OTUs (**a**); Coverage, *C* (**b**); Shannon index, *H*′ (**c**); and Simpson index, *D* (**d**), of the rhizosphere samples collected at different growth stages of the cotton varieties

**Fig. 3:**
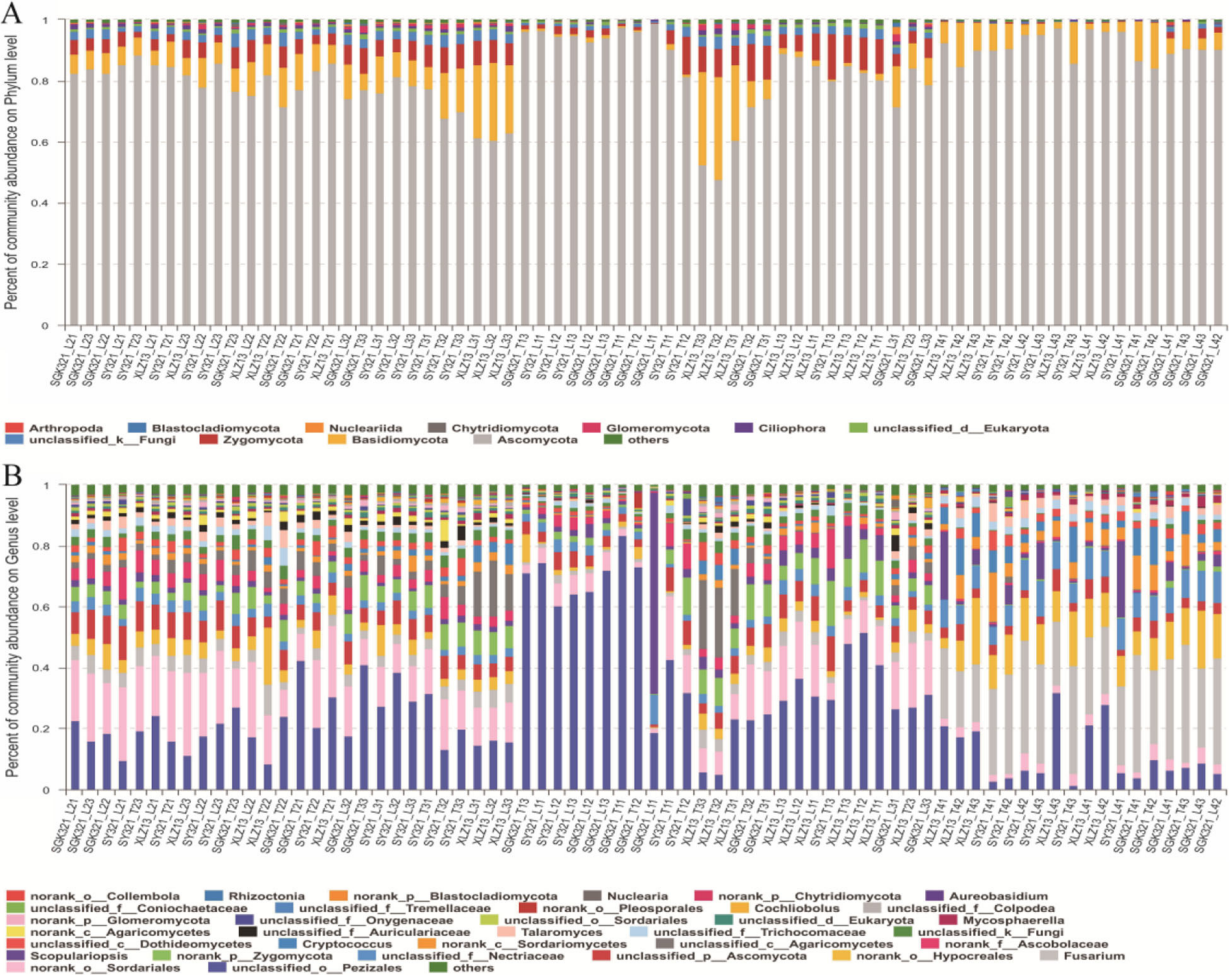
Relative abundances of different fungal phyla (**a**) and genera (**b**) in the rhizosphere communities

**Fig. 4:**
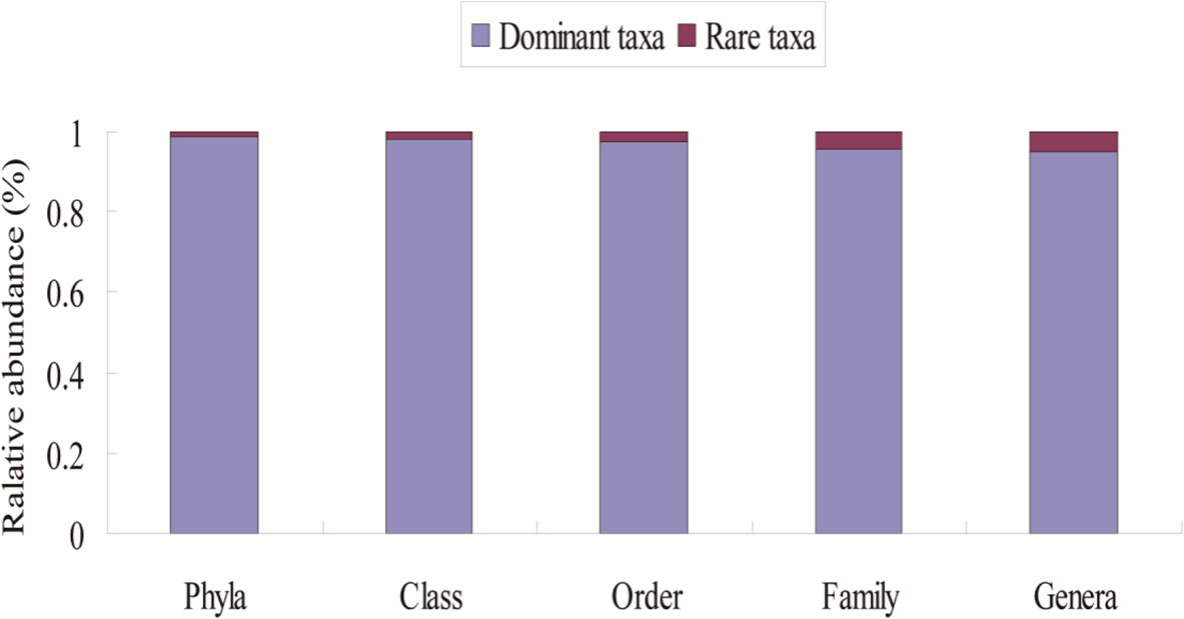
Relative abundances of sequence load of the dominant and rare taxa at different hierarchical ranks

A total of 1907,500 high-quality 18S rRNA gene sequences were generated by Illumina MiSeq sequencing of the 72 samples, with 16,635–35,015 sequences obtained per sample (Additional file 1: Table S2A, B, C, D). When clustered at the 97% nucleotide similarity level, 304 different phylotypes (OTUs) were present among the soils, and richness was significantly higher at the budding and flowering stages (Fig. 2a). OTU richness was significantly different between the taproot and lateral root rhizospheres of SGK321 at the seedling stage, but no significant differences were observed when comparing the same root zones between SGK321 and SY321. Moreover, there were no significant differences in OTU richness between different varieties and root zones at other stages. The 26 most abundant OTUs (> 0.5% relative abundance) comprised sequences from all of the 72 samples (Additional file 1: Table S3A, B, C, D), and 25 of these OTUs were present in all of the samples. Rarefaction curves of all soil samples reached their asymptotes, indicating that the data generated in this study was enough for the analysis of the diversity of fungi in the rhizosphere (Additional file 1: Figure S1). A total of 40 fungal phyla and 127 genera were identified throughout the growth stages, with the predominant phyla being *Ascomycota* (83.31%), *Basidiomycota* (7.81%), *Zygomycota* (4.40%), unclassified *Fungi* (1.96%), and unclassified *Eukaryota* (0.69%), among others. The predominant genera were unclassified *Pezizales* (27.28%), unclassified *Sordariales* (11.42%), *Fusarium* (9.29%), unclassified *Hypocreales* (5.90%), and unclassified *Ascomycota* (5.49%), among others (Fig. 3a, b). The six most abundant phyla (each accounting for > 0.05% of fungal sequences) represented 98.71% of the total sequence data, while the remaining 34 rare phyla comprised only 1.29% of the total sequence data. Likewise, the 21 most abundant genera represented 94.90% of the total sequence data, while the remaining 106 rare genera constituted only 5.10% of the total sequence data. Thus, the trend of few dominant taxa to the group with high sequence load and most of the rare taxa to the group with low sequence load could be found at different taxonomic ranks, and the relative sequence of rare taxa increased gradually at the lower hierarchical ranks (Fig. 4).

### Diversity of rhizosphere fungi

An ANOSIM test (*R*^2^ = 0.8092, *P* = 0.001) (Additional file 1: Figure S2) showed that the difference between the groups was significantly greater than that within the group, suggesting the results obtained from this study were reliable. The library coverage, *C*, based on OTU richness was > 99.93% (Fig. 2b), suggesting that the vast majority of all taxonomic units were detected in 72 samples, including in the sample SGK321_T41, that had the lowest number of sequences of any sample (16,635; 0.96%). The alpha diversity indices comprising the Shannon index *H*′ and Simpson index *D* of the fungal communities significantly differed among rhizosphere communities at the different growth stages of cotton (Fig. 2c, d). Specifically, *H*′ was significantly higher at the budding and flowering stages, while *D* was significantly higher at the seedling and bolling stages. However, the *H*′ and *D* values were not significantly different at any of the sampling time points when comparing the taproot and lateral root rhizospheres of SGK321, SY321, and XLZ13. Thus, these results indicate that the variation in diversity of fungal communities was determined by cotton growth stages but not by varieties, including *Bt* cotton SGK321.

### Effect of *Bt* cotton cultivation on dominant and rare fungal taxa

The richness and abundance of all of the genera, the dominant genera, and the rare genera subsets of the fungal communities changed significantly at the different growth stages (Table 1). The richness of all of the genera in the lateral root and taproot soil communities of SGK321 was significantly different between the seedling and bolling stages. However, comparisons of SGK321 and SY321 indicated no differences at the same stage for the same root zones, and similar results were observed for rare genera. Notably, the abundances of all of the genera, the dominant genera, and the rare genera were not different throughout the growth stages for the three cotton varieties. When compared with the richness and abundance of SY321 lateral root of different growth stages, the richness and abundance changes of all of the genera, the dominant genera, and the rare genera of fungal communities of different cotton varieties and root zones are shown in Table 2. The richness of rare genera and all of the genera correlated similarly to the cultivation of *Bt* cotton, suggesting that changes in fungal community richness were mainly due to the effects arising from the variety and the root zones on the richness of rare genera. In addition, similar progressions were observed for the abundance distribution of all of the genera and the dominant genera at different growth stages, indicating that the changes in fungal community abundances at different stages were mainly derived from the effects arising from variety type and the root zones on the abundances of dominant genera. Consequently, rare genus abundances were more responsive to external interference when compared to the dominant genera. In order to assess whether distinct taxa were preferred among the dominant genera, the class-level taxonomic assignments of the 21 dominant genera, 106 rare genera, and 120 responsive genera were compared with that of all of the 127 genera (Table 3). The proportion of contributing genera (> 7 genera of one class) among all of the genera was similar to that of the responsive genera for most classes. The dominant genera were generally preferred to associate with the *Sordariomycetes* and *Agaricomycetes*, while the rare genera were generally preferred to associate with the *Intramacronucleata*. In addition, the proportion of contributing genera belonging to the *Dothideomycetes* was in accordance with that of the dominant genera.

**Table 1:** Richness and abundances of total, dominant, and rare fungal genera subsets among different comparison treatments and at different growth stages (mean ± SE)

Stage	Variety	Total genera		Dominant genera		Rare genera	
		Richness	Abundance	Richness	Abundance	Richness	Abundance
Seedling	SGK321_L1	61.33 ± 8.02^d^	30,749.67 ± 272.49^ab^	16 ± 0^a^	29,797.00 ± 930.67^ab^	45.33 ± 8.08^bc^	952.67 ± 337.30^a^
	SGK321_T1	54.00 ± 4.58^e^	23,448 ± 2714.27^b^	16 ± 0^a^	22,481.00 ± 2984.87^b^	38.00 ± 4.58^d^	967.00 ± 371.73^a^
	SY321_L1	62.00 ± 3.46^cd^	33,984.67 ± 915.88^a^	16 ± 0^a^	32,767.67 ± 1054.13^a^	46.00 ± 3.46^bc^	1217.00 ± 138.79^a^
	SY321_T1	55.00 ± 2.00^de^	20,718 ± 1677.02^b^	16 ± 0^a^	19,782.67 ± 1615.04^b^	39.00 ± 2.00^cd^	935.33 ± 264.31 ^a^
	XLZ13_L1	66.33 ± 1.53^cd^	23,487 ± 3251.14^b^	16 ± 0^a^	22,096.00 ± 2947.17^b^	50.33 ± 1.53^bc^	1391.00 ± 335.56^a^
	XLZ13_T1	63.00 ± 1.73^cd^	25,711 ± 3779.24^b^	16 ± 0^a^	24,736.67 ± 3726.54^b^	47.00 ± 1.73^bc^	974.33 ± 310.55^a^
Budding	SGK321_L2	76.00 ± 3.61^b^	28,439.33 ± 6645.62^ab^	24 ± 0^a^	27,202.00 ± 6238.60^ab^	52.00 ± 3.60^b^	1237.33 ± 411.59^a^
	SGK321_T2	72.00 ± 4.36^bc^	28,891.67 ± 1289^ab^	24 ± 0^a^	27,519.33 ± 6768.36^ab^	48.00 ± 4.35^bc^	1372.00 ± 396.82^a^
	SY321_L2	68.67 ± 2.52^c^	29,648.33 ± 4692.02^ab^	24 ± 0^a^	28,472.33 ± 4519.09^ab^	44.67 ± 2.51^c^	1176.00 ± 228.91^a^
	SY321_T2	68.67 ± 5.77^c^	22,655.00 ± 7198.60^b^	24 ± 0^a^	21,759.33 ± 6768.36^b^	44.67 ± 5.77^c^	895.67 ± 458.45^a^
	XLZ13_L2	75.33 ± 6.51^bc^	32,332.33 ± 2572.90^ab^	24 ± 0^a^	30,900.00 ± 2466.60^ab^	51.33 ± 6.51^bc^	1432.33 ± 190.73^a^
	XLZ13_T2	76.00 ± 3.61^b^	28,161.33 ± 939.20^ab^	24 ± 0^a^	27,059.33 ± 949.56^ab^	52.00 ± 3.61^b^	1102.00 ± 139.11^a^
Flowering	SGK321_L3	80.00 ± 8.19^ab^	24,009.67 ± 2609.50^b^	24 ± 0^a^	22,621 ± 2918.30^b^	56.00 ± 8.19^ab^	1388.67 ± 310.67^a^
	SGK321_T3	75.33 ± 1.53^bc^	26,758.33 ± 6106.19^b^	24 ± 0^a^	25,382.00 ± 5736.58^b^	51.33 ± 1.53^bc^	1376.33 ± 392.33^a^
	SY321_L3	81.00 ± 4.36^ab^	28,499.33 ± 7956.49^a^	24 ± 0^a^	27,363.00 ± 7699.97^ab^	57.00 ± 4.36^ab^	1136.33 ± 257.71^a^
	SY321_T3	74.33 ± 5.51^bc^	27,820.00 ± 901.98^ab^	24 ± 0^a^	26,252.00 ± 1074.15^b^	50.33 ± 5.51^bc^	1568.00 ± 188.38^a^
	XLZ13_L3	75.67 ± 1.15^bc^	28,054.00 ± 3582.16^ab^	24 ± 0^a^	26,674.67 ± 3413.87^ab^	51.67 ± 1.15^bc^	1379.33 ± 168.30^a^
	XLZ13_T3	84.00 ± 3.46^a^	22,696.67 ± 3184.21^b^	24 ± 0^a^	21,420.33 ± 2990.36^b^	60.00 ± 3.46^a^	1276.33 ± 363.00^a^
Bolling	SGK321_L4	63.00 ± 4.36^cd^	22,356.00 ± 567.76^b^	16 ± 0^a^	21,147.67 ± 471.55^b^	47.00 ± 4.36^bc^	1208 ± 162.12^a^
	SGK321_T4	48.67 ± 3.21^e^	22,505.33 ± 5247.31^b^	16 ± 0^a^	21,725.00 ± 5443.78^b^	32.67 ± 3.12^d^	780.33 ± 209.92^a^
	SY321_L4	56.00 ± 2.65^de^	24,329.00 ± 2690.40^b^	16 ± 0^a^	23,682.33 ± 2677.12^b^	40.00 ± 2.65^cd^	646.67 ± 107.21^a^
	SY321_T4	59.67 ± 5.69^de^	25,403.33 ± 2294.66^b^	16 ± 0^a^	23,759.67 ± 2225.38^b^	43.67 ± 5.69^c^	1643.67 ± 549.72^a^
	XLZ13_L4	52.33 ± 2.08^e^	30,826.33 ± 1734.49^ab^	16 ± 0^a^	30,056.00 ± 1703.55^ab^	36.33 ± 2.08^d^	770.33 ± 114.51^a^
	XLZ13_T4	50.67 ± 2.89^e^	24,349.00 ± 5641.63^b^	16 ± 0^a^	23,628.33 ± 5581.22^b^	34.67 ± 2.89^d^	720.67 ± 418.25^a^

Different lowercase letters in the same column indicate significant differences at the 0.05 level

**Table 2:** Comparisons of the response values for genera richness and abundances among comparisons between varieties for total genera, dominant genera, and rare genera subsets (mean ± SE)

Stage	Variety/variety	Total genera		Dominant genera		Rare genera	
		Richness	Abundance	Richness	Abundance	Richness	Abundance
Seedling	SGK321_L1/SY321_L1	0.9892	0.9048	1.0000	0.9093	0.9855	0.7828
	SGK321_T1/SY321_L1	0.8700	0.6890	1.0000	0.6861	0.8261	0.7946
	XLZ13_L1/SY321_L1	1.0698	0.6096	1.0000	0.6743	1.0942	1.1430
	XLZ13_T1/SY321_L1	1.0161.	0.6911	1.0000	0.7549	1.0217	0.8006
	SY321_T1/SY321_L1	0.8871	0.7564	1.0000	0.6037	0.8478	0.7686
Budding	SGK321_L2/SY321_L2	1.1067	0.9592	1.0000	0.9554	1.1641	1.0522
	SGK321_T2/SY321_L2	1.0485	0.9745	1.0000	0.9665	1.0745	1.1667
	XLZ13_L2/SY321_L2	1.0970	1.0905	1.0000	1.0853	1.1492	1.2180
	XLZ13_T2/SY321_L2	1.1067	0.9498	1.0000	0.9504	1.1641	0.9371
	SY321_T2/SY321_L2	1.0000	0.7642	1.0000	0.7642	1.0000	0.7616
Flowering	SGK321_L3/SY321_L3	0.9877	0.8425	1.0000	0.8267	0.9825	1.2221
	SGK321_T3/SY321_L3	0.9300	0.9389	1.0000	0.9276	0.9006	1.2112
	XLZ13_L3/SY321_L3	0.9342	0.9844	1.0000	0.9748	0.9043	1.2138
	XLZ13_T3/SY321_L3	1.0370	0.7964	1.0000	0.7828	1.0526	1.1232
	SY321_T3/SY321_L3	0.9177	0.9762	1.0000	0.9594	0.8830	1.3799
Bolling	SGK321_L4/SY321_L4	1.1250	0.9189	1.0000	0.8930	1.1750	1.8685
	SGK321_T4/SY321_L4	0.8691	0.9250	1.0000	0.9173	0.8167	1.2067
	XLZ13_L4/SY321_L4	0.9345	1.2671	1.0000	1.2691	0.9083	1.1912
	XLZ13_T4/SY321_L4	0.9048	1.0008	1.0000	0.9977	0.8667	1.1144
	SY321_T4/SY321_L4	1.0655	1.0442	1.0000	1.0032	1.0917	2.5417

**Table 3:** Number of genera that were assigned to different classes with respect to their relative abundances or significant differential response to different root environment treatments. Absolute number of genera (#) and the proportion of all of the genera in the respective category (%) are given for the different categories

Class	All genera		Dominant genera		Rare genera		Responsive genera	
	#	%	#	%	#	%	#	%
Intramacronucleata Alveolata_c_norank_k__Alveolata MPE1–14 Flabellinia Longamoebia Gracilipodida_c_norank_p__Gracilipodida LKM74_c_norank_p__LKM74 Schizoplasmodiida_c_norank_p__Schizoplasmodiida Euamoebida Leptomyxida WIM_1_lineage WIM5_c_norank_p__WIM5 Amoebozoa_c_unclassified_k__Amoebozoa Acanthocystidae_c_norank_p__Acanthocystidae Centrohelida_c_norank_k__Centrohelida Centrohelida_c_unclassified_k__Centrohelida Embryophyta Chloroplastida_c_norank_k__Chloroplastida Nucleariidae Discicristoidea_c_norank_k__Discicristoidea Dothideomycetes Eurotiomycetes Orbiliomycetes Pezizomycetes Saccharomycetes Sordariomycetes Ascomycota_c_norank_p__Ascomycota Ascomycota_c_unclassified_p__Ascomycota Agaricomycetes Agaricostilbomycetes Exobasidiomycetes Microbotryomycetes Pucciniomycetes Tremellomycetes Basidiomycota_c_norank_p__Basidiomycota Blastocladiomycota_c_norank_p__Blastocladiomycota Chytridiomycota_c_norank_p__Chytridiomycota Cryptomycota_c_norank_p__Cryptomycota Glomeromycota_c_norank_p__Glomeromycota Zygomycota_c_norank_p__Zygomycota Fungi_c_norank_k__Fungi Fungi_c_unclassified_k__Fungi Clitellata Arachnida Ellipura Insecta Chromadorea Glissomonadida Imbricatea RM2-SGM58 Vampyrellidae Cercozoa_c_norank_p__Cercozoa Cercozoa_c_unclassified_p__Cercozoa Bicosoecida_c_norank_p__Bicosoecida Bicosoecida_c_unclassified_p__Bicosoecida Hyphochytriomycetes_c_norank_p__Hyphochytriomycetes Chrysophyceae Peronosporomycetes_c_norank_p__Peronosporomycetes Craspedida Choanomonada_c_unclassified_p__Choanomonada Ichthyophonae LKM15_c_norank_p__LKM15 Preaxostyla_c_norank_p__Preaxostyla Eukaryota_c_unclassified_p__norank_d__Eukaryota Eukaryota_c_unclassified_d__Eukaryota	10 1 1 1 1 2 1 2 1 1 1 1 1 1 1 1 2 1 1 1 10 4 1 3 3 15 1 1 7 1 3 1 2 4 1 1 1 1 1 1 1 1 2 1 1 2 2 1 1 1 1 1 1 1 1 1 2 1 4 1 2 1 1 1 1	7.87 0.79 0.79 0.79 0.79 1.57 0.79 1.57 0.79 0.79 0.79 0.79 0.79 0.79 0.79 0.79 1.57 0.79 0.79 0.79 7.87 3.15 0.79 2.36 2.36 11.81 0.79 0.79 5.51 0.79 2.36 0.79 1.57 3.15 0.79 0.79 0.79 0.79 0.79 0.79 0.79 0.79 1.57 0.79 0.79 1.57 1.57 0.79 0.79 0.79 0.79 0.79 0.79 0.79 0.79 0.79 1.57 0.79 3.15 0.79 1.57 0.79 0.79 0.79 0.79	0 0 0 0 0 0 0 0 0 0 0 0 0 0 0 0 0 0 0 0 2 2 0 2 0 7 0 1 3 0 0 0 0 1 0 0 0 0 0 1 0 1 0 0 0 0 0 0 0 0 0 0 0 0 0 0 0 0 0 0 0 0 0 0 1	0 0 0 0 0 0 0 0 0 0 0 0 0 0 0 0 0 0 0 0 9.52 9.52 0 9.52 0 33.33 0 4.76 14.29 0 0 0 0 4.76 0 0 0 0 0 4.76 0 4.76 0 0 0 0 0 0 0 0 0 0 0 0 0 0 0 0 0 0 0 0 0 0 4.76	10 1 1 1 1 2 1 2 1 1 1 1 1 1 1 1 2 1 1 1 8 2 1 1 3 8 1 0 4 1 3 1 2 3 1 1 1 1 1 0 1 0 2 1 1 2 2 1 1 1 1 1 1 1 1 1 2 1 4 1 2 1 1 1 0	9.43 0.94 0.94 0.94 0.94 1.89 0.94 1.89 0.94 0.94 0.94 0.94 0.94 0.94 0.94 0.94 1.89 0.94 0.94 0.94 7.55 1.89 0.94 0.94 2.83 7.55 0.94 0 3.77 0.94 2.83 0.94 1.89 2.83 0.94 0.94 0.94 0.94 0.94 0 0.94 0 1.89 0.94 0.94 1.89 1.89 0.94 0.94 0.94 0.94 0.94 0.94 0.94 0.94 0.94 1.89 0.94 3.77 0.94 1.89 0.94 0.94 0.94 0	10 0 1 1 1 1 1 2 1 1 1 1 1 1 1 1 2 1 1 1 10 4 1 3 3 15 1 1 6 1 2 1 2 4 1 1 1 1 1 1 1 1 2 1 1 2 1 1 1 1 1 0 1 1 1 1 2 1 3 1 2 1 1 1 1	8.33 0 0.83 0.83 0.83 0.83 0.83 1.67 0.83 0.83 0.83 0.83 0.83 0.83 0.83 0.83 1.67 0.83 0.83 0.83 8.33 3.33 0.83 2.50 2.50 12.5 0.83 0.83 5.00 0.83 1.67 0.83 1.67 3.33 0.83 0.83 0.83 0.83 0.83 0.83 0.83 0.83 1.67 0.83 0.83 1.67 0.83 0.83 0.83 0.83 0.83 0 0.83 0.83 0.83 0.83 1.67 0.83 2.50 0.83 1.67 0.83 0.83 0.83 0.83

### Cluster analysis of rhizosphere fungal communities of different roots environment

**Fig. 5:**
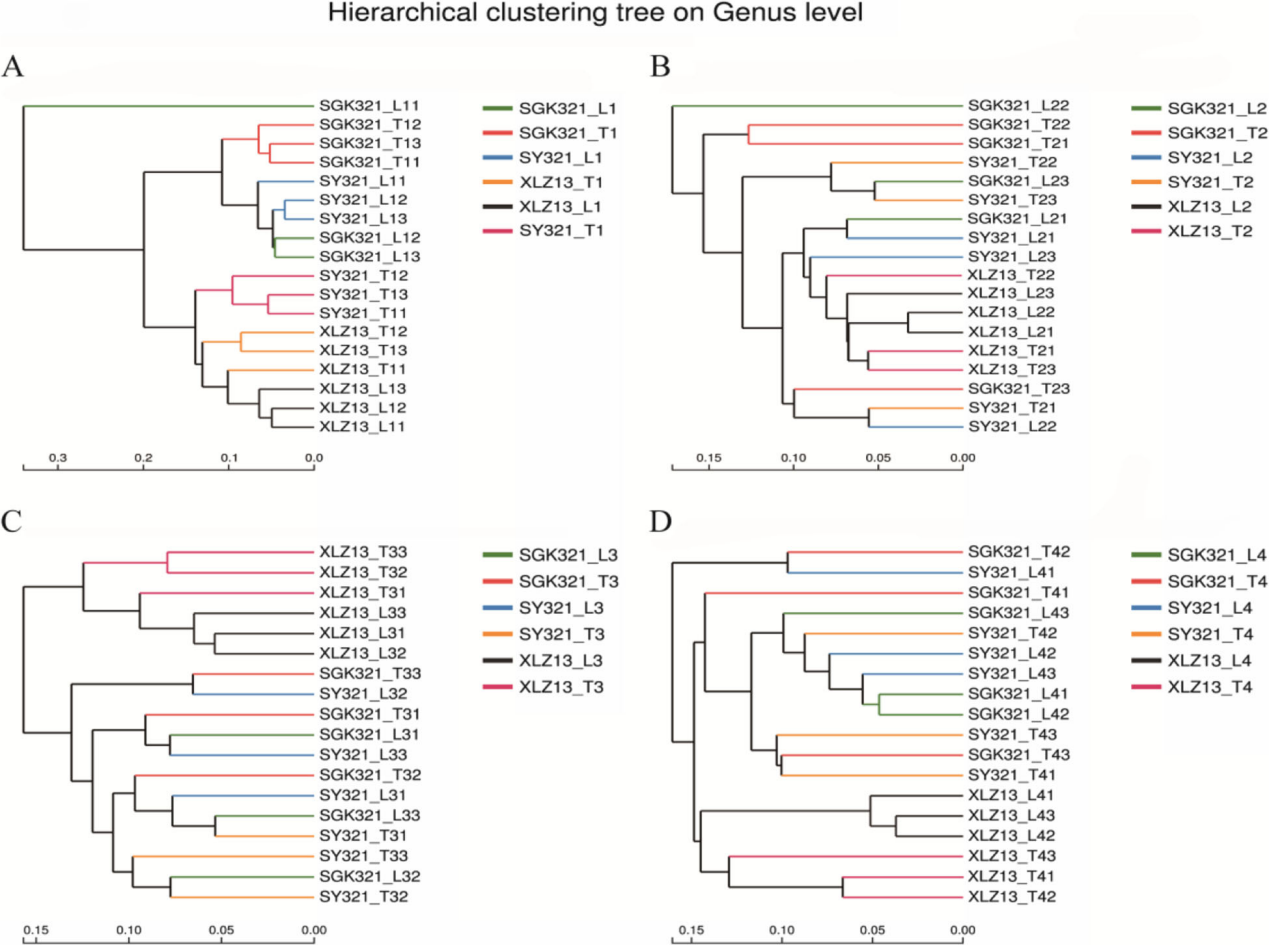
Hierarchical cluster analysis of the fungal communities from the rhizosphere of different cotton varieties and root environments at seedling (**a**), budding (**b**), flowering (**c**), and bolling (**d**) growth stages. Distances are based on the total fungal genera and the Hellinger distances between microbial communities

Cluster analyses of the relative abundances of all of the genera indicated that the lateral root and taproot rhizospheres of the conventional variety XLZ13 clustered together. These results highlighted the distinct fungal community compositions of XLZ13 compared to rhizospheres from the GM variety (SGK321) or the parental cotton variety (SY321) (Fig. 5). Separation of fungal communities from lateral roots or taproots was observed at the seedling stage of the three varieties, suggesting an impact of the root environment on fungal community composition. The lack of similar separation at other growth stages suggests that fungal communities were also affected by other factors, such as climate or seasonality, for example. Rhizosphere samples from SGK321 and SY321 were highly similar, and communities from all of the growth stages clustered closely. Thus,* Bt* cotton, such as SGK321, did not have specific effects on fungal community composition. When cluster analyses of the dominant, rare, and responsive genera subsets were conducted (Additional file 1: Figures S3-S5), similar clustering patterns were observed between the dominant and all of the genera subsets, suggesting that the dominant genera primarily contributed to the overall composition dynamics of the communities. In contrast, differences based on the rare genera subset resulted in clustering that was highly distinct from the clustering based on all of the genera. The fungal communities of the soil samples based on the responsive genera clustered roughly into six groups corresponding to different treatments (cotton varieties and root zones). A total of 120 genera significantly responded in its abundance to one variety in comparison with its abundance in all other varieties or to the root environment at different growth stages.

### Relationship between rhizosphere fungal community composition and soil pH and Bt protein

**Fig. 6:**
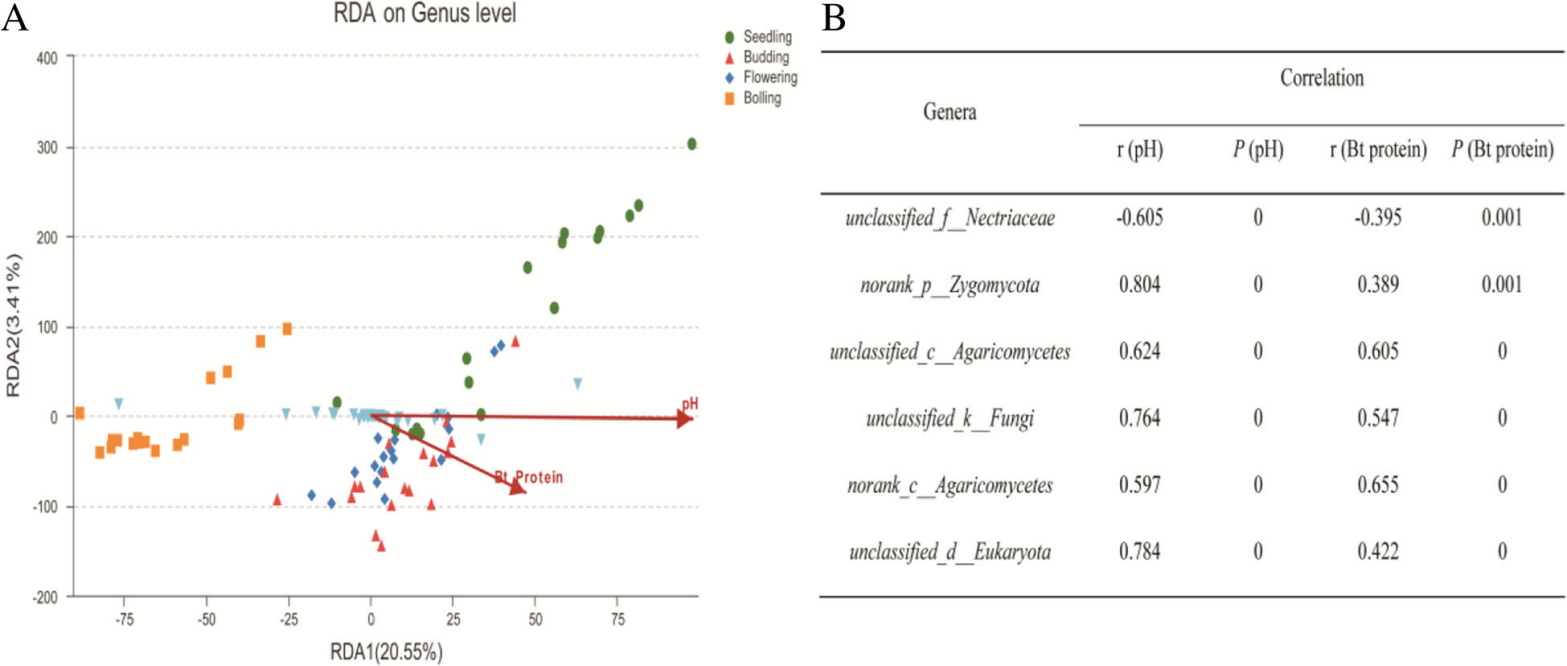
RDA of fungal community compositional differences in relation to pH and Bt protein content at different growth stages of the cotton varieties (**a**). Partial data of the Spearman’s rank correlations between fungal genera and soil pH and Bt protein content (**b**). All of the analyses were conducted in triplicate

The relationship between fungal community composition and soil pH and Bt protein content was analyzed by RDA (Fig. 6a). The first two axes of the RDA accounted for 23.96% of the total variance in fungal community composition, with the first axis accounting for 20.55% of the variance. The distribution pattern of the samples from the seedling and bolling stages was discrete, while that at the budding and flowering stages clustered together. Soil pH was more correlated to community composition than soil Bt protein content, suggesting more important effects arising from soil pH. This conclusion was also supported by a Mantel test, indicating that soil pH (*r* = 0.700, *P* = 0.001) was more correlated to fungal community composition than Bt protein content (*r* = 0.417, *P* = 0.001). We also used Spearman’s rank order correlations to investigate the response of specific fungal genera over time to soil pH and Bt protein content (Fig. 6b). The abundances of unclassified *Zygomycota* (*r* = 0.804, *P* = 0.001), unclassified *Fungi* (*r* = 0.764, *P* = 0), and unclassified *Eukaryota* (*r* = 0.784, *P* = 00) were positively correlated with soil pH. In contrast, unclassified *Nectriaceae* (*r* = − 0.605, *P* = 0.001) abundances were negatively correlated with soil pH. Lastly, the abundances of no rank *Agaricomycetes* (*r* = 0.655, *P* = 0) and unclassified *Agaricomycetes* (*r* = 0.605, *P* = 0) were positively correlated with soil Bt protein content.

## Discussion

### Differences in the variation of dominant and rare fungal taxa in relation to the cultivation of *Bt* and conventional cotton varieties

Microorganisms present in the rhizosphere are affected by root exudates and play important roles in the growth and ecological fitness of their plant host. Rhizosphere microorganisms are considered to be an important component of soil ecological system. Consequently, the microbial community and their associated dynamic processes are the foundations of ecosystems. However, the importance of rare, low-abundance microorganisms is largely unknown, although it is hypothesized that they contribute to the community stability by acting as a reservoir that can rapidly respond to environmental changes [31]. Fungi are ubiquitous microorganisms that play important roles in soil ecosystems as major decomposers of organic matter, and they release nutrients during nutrient cycling that stimulate plant growth [32]. Thus, it is critical to assess the contribution of dominant and rare fungi toward root exudates including Bt protein in light of the diversity and processes of fungal communities. To explore this issue, we employed deep sequencing of uncultured fungal communities to investigate the response of less abundant, rare fungal community members to the cultivation of *Bt* cotton plants.

Here, the community structure of the dominant genera obtained the similar clustering pattern with all genera throughout all the growth stages, which is similar to the variation of the bacterial community [4]. In contrast, the communities represented by the rare genera clustered differently, and this was consistent with the community clustering due to the responsive genera. Previous researches have investigated microbial indicators for the purpose of evaluating correlations between microbes and for identifying possible sources of specific environment variation [33–35]. However, the majority of these researches lacked sampling power to comprehensively identify the indicators and assess the dynamic nature of indicators due to the use of low-throughput sequencing. We used high-throughput sequencing on the Illumina platform to better understand the rare fungal taxa, which are more sensitive to disturbances. Only 20 of the 120 responsive genera identified in this study were dominant genera, whereas 100 were rare. The detection of responsive genera associated with *Bt* cotton cultivation provides targets to identify the fungal taxa potentially responsible for degrading and utilizing Bt protein in the rhizosphere. Furthermore, our analyses revealed responsive fungal taxa that may be vulnerable to environmental change, with cotton plant varieties and/or root zones acting as proxies for environmental differences. These results suggest that some of these taxa may be indicators of unmeasured environmental changes and thus provide targets to assess biological drivers that are responsive to subtle physical, chemical, or biological changes. Finally, we found that the response of dominant genera abundances to differences in cotton varieties and/or root zones was consistent with that of all of the genera at different growth stages, while the abundances of rare genera displayed distinctive clustering trends. These results indicate that changes of abundance in the fungal community were caused by the effects of aforementioned treatments on the dominant genera. Indeed, the abundances of rare genera were markedly susceptible to environmental changes, which are similar to the observations from Shade et al. [31]. Compared to the remarkable change of abundances throughout the growth stages, the richness of dominant genera changed only slightly. Further, the variation in richness values for the rare and all of the genera subsets was similar, suggesting that changes in community richness were mainly due to the effects of treatments on the rare genera. Although this study indicated the importance of dominant and rare taxa for microbial diversity in the rhizosphere of *Bt* and conventional cotton varieties, the metabolic potentials and ecological roles of rhizosphere dominant and rare microbes to the consecutive cultivation of *Bt* cotton revealed by metagenomics and metatranscriptomics need to be further analyzed in a subsequent experiment.

### Differences in fungal community structure with different root zones and possible factors regulating fungal distribution in roots

The plant rhizosphere is a dynamic environment in which many factors may affect the structure and species composition of microbial communities that colonize the roots [20]. These factors can include host plant species [36] or cultivars [37] grown in the same soil, and even different root zones of the same plant [38]. Here, we sampled lateral roots and taproots at all of the sampling times, and we collected soils in triplicate in order to minimize sample variation but reflect overall differences associated with root zones. The abundances of ITS rRNA gene copies were clearly affected by (i) cotton species, (ii) growth stages, and (iii) root zones. In particular, the lateral root of SGK321 exhibited lower fungal abundances than taproots. Notably, Zhang et al. [39] reported that the rhizosphere had a lower fungal diversity (observed OTUs and Chao1) than bulk soil and a distinct fungal community structure in the rhizosphere compared with bulk soil. In this study, during the periods of seedling, budding, and flowering stages, fungal abundance of rhizosphere soil revealed higher than that of bulk soil, but the fungal abundance decreased in rhizosphere soil at bolling stage, without a significant difference with that in bulk soil. These results are consistent with those of Marchand et al. [40], who found that different microbial population were obtained in rhizosphere and bulk soils during the vegetative growth phase, but there is no significant difference of diversity of culturable microflora between rhizosphere and the bulk soil during the reproductive growth stage. Consequently, in the present study, we speculated that the remarkable decrease of root exudates due to the decline of root function at the bolling stage is unable to alter soil fungal abundance significantly. Moreover, PCA at OTU level (Additional file 1: Figure S6) showed that the fungal community structure in rhizosphere soil had significant differences compared with the bulk soil at the different growth stages. In this study, cluster analysis indicated that the conventional variety, XLZ13, had a different fungal community composition compared to the *Bt* cotton variety, SGK321, or its conventional parental variety, SY321, throughout the various growth stages. These results suggest that the variation introduced by different conventional cotton varieties might be greater than those between transgenic and conventional cotton varieties. Moreover, lateral roots or taproots of the three cotton varieties grouped separately at the seedling stage, indicating that the rhizosphere fungal community was highly influenced by root microhabitats during this growth period. Importantly, previous studies centered on *Bt* cotton [22, 40–42], and it is indispensable to assess and monitor the impacts of *Bt* cotton cultivation on agroecosystems according to the case-by-case principle of biosafety assessment. Thus, to thoroughly assess the effects of genetically modified dicotyledons (cotton, soybean, rapeseed, among others) on soil microbial communities, particular root zones should be considered carefully, because they play significant roles in determining the quantitative and qualitative composition of exudates. Badri and Vivanco [19] suggested that root-secreted chemicals mediate multi-partite interactions in the rhizosphere and that root exudates initiate and modulate dialog between roots and soil microorganisms. Thus, we hypothesized that wide variation in the quantity and quality of root exudates, including Bt protein content, between lateral roots and taproots might affect the dynamics of microbial communities, including nutrient cycling. Consequently, the effect of organic compounds produced in the lateral roots and taproots on selecting microbial community members warrants further investigation. It should also be noted that different fungal populations between the taproots and lateral roots may play specific roles in the different root zones and in the overall ecological fitness of their plant host.

### Cultivation of *Bt* cotton alters Bt protein content and soil pH associated with shifts in soil fungal communities

Recent research has demonstrated that Bt proteins encoded by *cry* genes from *Bacillus thuringiensis* are released in root exudates from *Bt* plants [43, 44]. However, it is not yet clear whether Bt proteins in root exudates significantly influence soil microbial communities. In this study, the Bt protein content from SGK321 was significantly higher than that of SY321 and XLZ13 when considering the same root zones. Notably, this study observed differences of Bt protein from the lateral roots and taproots of Bt cotton at the seedling and budding stages for the first time. These differences in Bt protein content may be due to the differences in Bt protein content within the cotton taproot and lateral root tissues. Hence, we measured Bt protein content in different root tissues zones. However, the values were not significantly different between the taproot and lateral root tissues (Additional file 1: Figure S7). Parker et al. [45] described that root hairs comprised as much as 77% of the total root surface area of cultivated crops, forming the major point of contact between the plant and rhizosphere. Accordingly, the enlarged interface between cotton and rhizosphere due to the presence of more root hairs in the lateral roots may result in marked alterations of rhizosphere Bt protein contents in different root location. Although RDA indicated that the soil fungal communities were significantly correlated to Bt protein content (range, 54.43 to 303.94 pg/g soil; *r* = 0.417, *P* = 0.001) through all of the growth stages, the correlation was not significant when analyzed for individual growth stages. This finding implies that the shifts in the composition of soil fungal communities were due to the effects of the cotton growth stage rather than the variation in Bt protein content. Soil pH is one of the most influential chemical factors affecting soil microbial communities, as it affects numerous abiotic factors, including carbon and nutrient availability [46]. To explain the pH variability within the rhizosphere between SGK321 and SY321 (or XLZ13) at the seedling and budding stages, we cultivated the varieties in sterile Hoagland’s nutrient solution. The total amount of organic acids in root exudates of SGK321 was dramatically lower than that of SY21 and XLZ13 (Additional file 1: Table S4). Thus, the higher rhizosphere pH value of SGK321 might be related to pronounced organic acid decreases in the root exudates, in comparison to the control varieties. Further, soil pH was the strongest predictor of soil fungal community composition, followed closely by Bt protein content, suggesting that Bt protein introduced by root exudates did not impact fungal community composition more strongly than soil pH. Notably, several studies have shown that Bt protein expressed by *Bt* crops does not persist in the soil after long-term cultivation of *Bt* crops [10, 47, 48]. Moreover, environmental factors and cotton growth traits are specific at different growth stages, which affect the diversity and dynamics of rhizosphere microbial communities differently. Accordingly, we deduced that these findings obtained from rhizosphere and bulk soil samples at four different growth stages of cotton in this study were probably consistent with the research of the longer-term cultivation of *Bt* cotton.

## Conclusions

In summary, we monitored the diversity and dynamics of the rhizosphere fungal community from lateral roots and taproots of *Bt* plants for the first time using qPCR and high-throughput sequencing approaches. The dominant and rare fungal community taxa differentially responded to the cultivation of *Bt* cotton. Furthermore, fungal community structure was substantially affected by different root zones at the seedling stage, while the *Bt* cotton cultivar had no specific effect on fungal diversity in comparison to the conventional cotton varieties. Our results indicate that soil pH more strongly affected fungal community structure compared to soil Bt protein content. However, further investigations are needed to assess the variation in the organic compound composition of root exudates that were observed between the lateral roots and taproots of the *Bt* cotton variety and the consequent interactions between root exudates and fungal communities.

## Additional file

Additional file 1: **Table S1**. Terminology of rhizosphere samples included in this study. **Table S2. ** Number of sequences obtained per soil sample. Seedling (A), Budding (B), Flowering (C) and Bolling (D) stages. **Table S3.** Most abundant OTUs (> 0.5% relative abundance) shared by sequences of at least 97% sequence similarity. Seedling (A), Budding (B), Flowering (C) and Bolling (D) stages. **Table S4.** Types and quantities of the secreted organic acids in the culture solution (μg/d) (*n* = 3). **Figure S1.** Rarefaction curves of all of the rhizosphere samples based on OTUs. **Figure S2.** Analysis of similarities (ANOSIM) for fungal communities of the rhizosphere samples based on OTUs. **Figure S3.** Hierarchical cluster analysis of the dominant fungal genera from the rhizosphere of different cotton varieties and root environments at seedling (A), budding (B), flowering (C), and bolling (D) stages, based on the Hellinger distances of microbial communities. **Figure S4.** Hierarchical cluster analysis of the rare fungal genera from the rhizosphere of different cotton varieties and root environments at seedling (A), budding (B), flowering (C), and bolling (D) stages, based on the Hellinger distances of microbial communities. **Figure S5.** Hierarchical cluster analysis of the responsive fungal genera from the rhizosphere of different cotton varieties and root environments at seedling (A), budding (B), flowering (C), and bolling (D) stages, based on the Hellinger distances of microbial communities. **Figure S6.** PCA analysis of fungal community at seedling (A), budding (B), flowering (C), and bolling (D) stages based on OTU level. BS indicates bulk soil control. **Figure S7.** Bt protein contents of the different root tissues collected at different growth stages of the cotton varieties. (DOC 5941 kb)
