# Supplementary material for: The response of dominant and rare taxa for fungal diversity within different root environments to the cultivation of Bt and conventional cotton varieties
Source: Microbiome. 2018 Oct 18;6:184. doi: 10.1186/s40168-018-0570-9 (PMC6194802; doi:10.1186/s40168-018-0570-9)
Supplement: Supplementary file 1 — Table S1. Terminology of rhizosphere samples included in this study. Table S2. Number of sequences obtained per soil sample. Seedling (A), Budding (B), Flowering (C) and Bolling (D) stages. Table S3. Most abundant OTUs (> 0.5% relative abundance) shared by sequences of at least 97% sequence similarity. Seedling (A), Budding (B), Flowering (C) and Bolling (D) stages. Table S4. Types and quantities of the secreted organic acids in the culture solution (μg/d) (n = 3). Figure S1. Rarefaction curves of all of the rhizosphere samples based on OTUs. Figure S2. Analysis of similarities (ANOSIM) for fungal communities of the rhizosphere samples based on OTUs. Figure S3. Hierarchical cluster analysis of the dominant fungal genera from the rhizosphere of different cotton varieties and root environments at seedling (A), budding (B), flowering (C), and bolling (D) stages, based on the Hellinger distances of microbial communities. Figure S4. Hierarchical cluster analysis of the rare fungal genera from the rhizosphere of different cotton varieties and root environments at seedling (A), budding (B), flowering (C), and bolling (D) stages, based on the Hellinger distances of microbial communities. Figure S5. Hierarchical cluster analysis of the responsive fungal genera from the rhizosphere of different cotton varieties and root environments at seedling (A), budding (B), flowering (C), and bolling (D) stages, based on the Hellinger distances of microbial communities. Figure S6. PCA analysis of fungal community at seedling (A), budding (B), flowering (C), and bolling (D) stages based on OTU level. BS indicates bulk soil control. Figure S7. Bt protein contents of the different root tissues collected at different growth stages of the cotton varieties. (DOC 5941 kb) [file 40168_2018_570_MOESM1_ESM.doc]

Table S1. Terminology of samples analysed in experiment.

| Lateral  Taptoot | SGK321 | SY321 | XLZ13 |
| --- | --- | --- | --- |
| Seedling | SGK321_L11; SGK321_L12; SGK321_L13  SGK321_T11; SGK321_T12; SGK321_T13 | SY321_L11, SY321_L12, SY321_L13  SY321_T11, SY321_T12, SY321_T13 | XLZ13_L11, XLZ13_L12, XLZ13_L13  XLZ13_T11, XLZ13_T12, XLZ13_T13 |
| Budding | SGK321_L21; SGK321_L22; SGK321_L23  SGK321_T21; SGK321_T22; SGK321_T23 | SY321_L21, SY321_L22, SY321_L23  SY321_T21, SY321_T22, SY321_T23 | XLZ13_L21, XLZ13_L22, XLZ13_L23  XLZ13_T21, XLZ13_T22, XLZ13_T23 |
| Flowering | SGK321_L31; SGK321_L32; SGK321_L33  SGK321_T31; SGK321_T32; SGK321_T33 | SY321_L31, SY321_L32, SY321_L33  SY321_T31, SY321_T32, SY321_T33 | XLZ13_L31, XLZ13_L32, XLZ13_L33  XLZ13_T31, XLZ13_T32, XLZ13_T33 |
| Bolling | SGK321_L41; SGK321_L42; SGK321_L43  SGK321_T41; SGK321_T42; SGK321_T43 | SY321_L41, SY321_L42, SY321_L43  SY321_T41, SY321_T42, SY321_T43 | XLZ13_L41, XLZ13_L42, XLZ13_L43  XLZ13_T41, XLZ13_T42, XLZ13_T43 |

Table S2. (a) Number of sequences obtained per soil sample. Seedling (A), Budding (B), Flowering (C) and Bolling (D) stages.

(A)

| Number of sequences | SGK321_L11 | SGK321_L12 | SGK321_L13 | SGK321_T11 | SGK321_T12 | SGK321_T13 | SY321_L11 | SY321_L12 | SY321_L13 | SY321_T11 | SY321_T12 | SY321_T13 | XLZ13_L11 | XLZ13_L12 | XLZ13_L13 | XLZ13_T11 | XLZ13_T12 | XLZ13_T13 |
| --- | --- | --- | --- | --- | --- | --- | --- | --- | --- | --- | --- | --- | --- | --- | --- | --- | --- | --- |
| High quality  Percent (%) | 31045  1.79 | 30696  1.77 | 30508  1.76 | 21648  1.25 | 22126  1.27 | 17555  1.16 | 35015  2.02 | 33263  1.92 | 33676  1.94 | 22644  1.30 | 19929  1.15 | 19581  1.13 | 23953  1.38 | 20028  1.15 | 26480  1.52 | 24619  1.42 | 29916  1.72 | 22598  1.30 |

(B)

| Number of sequences | SGK321_L21 | SGK321_L22 | SGK321_L23 | SGK321_T21 | SGK321_T22 | SGK321_T23 | SY321_L21 | SY321_L22 | SY321_L23 | SY321_T21 | SY321_T22 | SY321_T23 | XLZ13_L21 | XLZ13_L22 | XLZ13_L23 | XLZ13_T21 | XLZ13_T22 | XLZ13_T23 |
| --- | --- | --- | --- | --- | --- | --- | --- | --- | --- | --- | --- | --- | --- | --- | --- | --- | --- | --- |
| High quality  Percent (%) | 27063  1.78 | 20781  1.37 | 17703  1.17 | 31482  1.81 | 17837  1.03 | 31611  1.82 | 23605  1.56 | 32869  1.89 | 24265  1.40 | 30930  1.78 | 25460  1.68 | 27032  1.78 | 17613  1.16 | 32586  1.88 | 29642  1.71 | 28926  1.67 | 28445  1.64 | 27113  1.56 |

(C)

| Number of sequences | SGK321_L31 | SGK321_L32 | SGK321_L33 | SGK321_T31 | SGK321_T32 | SGK321_T33 | SY321_L31 | SY321_L32 | SY321_L33 | SY321_T31 | SY321_T32 | SY321_T33 | XLZ13_L31 | XLZ13_L32 | XLZ13_L33 | XLZ13_T31 | XLZ13_T32 | XLZ13_T33 |
| --- | --- | --- | --- | --- | --- | --- | --- | --- | --- | --- | --- | --- | --- | --- | --- | --- | --- | --- |
| High quality  Percent (%) | 21403  1.23 | 24004  1.38 | 26622  1.53 | 19740  1.14 | 29682  1.71 | 30853  1.78 | 33071  1.90 | 33115  1.91 | 19312  1.11 | 27271  1.57 | 28861  1.66 | 27328  1.57 | 31909  1.84 | 27425  1.58 | 24828  1.43 | 24716  1.42 | 24348  1.40 | 19026  1.10 |

(D)

| Number of sequences | SGK321_L41 | SGK321_L42 | SGK321_L43 | SGK321_T41 | SGK321_T42 | SGK321_T43 | SY321_L41 | SY321_L42 | SY321_L43 | SY321_T41 | SY321_T42 | SY321_T43 | XLZ13_L41 | XLZ13_L42 | XLZ13_L43 | XLZ13_T41 | XLZ13_T42 | XLZ13_T43 | All samples |
| --- | --- | --- | --- | --- | --- | --- | --- | --- | --- | --- | --- | --- | --- | --- | --- | --- | --- | --- | --- |
| High quality  Percent (%) | 22562  1.30 | 21714  1.25 | 22792  1.31 | 16635  0.96 | 24141  1.39 | 26740  1.54 | 27226  1.57 | 23852  1.37 | 21909  1.26 | 27646  1.59 | 25504  1.47 | 23060  1.33 | 28924  1.67 | 32320  1.86 | 31235  1.80 | 30299  1.74 | 23671  1.36 | 19077  1.10 | 1736404 |

Table S3. Most abundant OTUs (>0.5% relative abundance) shared by sequences of at least 97% sequence similarity. Seedling (A), Budding (B), Flowering (C) and Bolling (D) stages.

(A)

| OTUs No. | Description | Sequences of each sample which were represented by the OTUs | | | | | | | | | | | | | | | | | |
| --- | --- | --- | --- | --- | --- | --- | --- | --- | --- | --- | --- | --- | --- | --- | --- | --- | --- | --- | --- |
|  |  | SGK321_L11 | SGK321_L12 | SGK321_L13 | SGK321_T11 | SGK321_T12 | SGK321_T13 | SY321_L11 | SY321_L12 | SY321_L13 | SY321_T11 | SY321_T12 | SY321_T13 | XLZ13_L11 | XLZ13_L12 | XLZ13_L13 | XLZ13_T11 | XLZ13_T12 | XLZ13_T13 |
| 98  76  304  86  91  241  152  251  153  298  286  124  302  1  300  186  103  14  200  203  128  303  64  227  242  190 | *unclassified_o__Pezizales norank_o__Sordariales*  *Fusarium*  *norank_o__Hypocreales*  *Scopulariopsis*  *norank_p__Zygomycota*  *unclassified_p__Ascomycota*  *norank_f__Ascobolaceae*  *norank_c__Sordariomycetes*  *unclassified_f__Nectriaceae*  *unclassified_c__Agaricomycetes*  *unclassified_c__Dothideomycetes*  *Cryptococcus*  *unclassified_o__Pezizales*  *unclassified_f__Nectriaceae*  *unclassified_f__Trichocomaceae*  *Talaromyces*  *norank_o__Sordariales*  *unclassified_p__Ascomycota*  *unclassified_f__Auriculariaceae norank_c__Agaricomycetes*  *Mycosphaerella*  *unclassified_p__Ascomycota*  *unclassified_o__Pezizales*  *unclassified_d__Eukaryota*  *unclassified_k__Fungi* | 3317  345  68  95  20594  83  159  144  48  641  8  48  5  2345  2373  40  28  37  96  16  3  18  25  173  20  37 | 18249  1444  477  272  1376  696  463  669  215  289  35  286  14  1016  700  274  140  385  315  213  36  77  206  252  177  50 | 20517  1373  241  243  449  666  526  782  117  147  20  198  20  1040  476  465  67  485  316  12  124  114  275  300  276  61 | 17365  300  143  378  201  167  94  157  68  85  3  172  15  529  393  88  25  176  235  7  32  35  217  197  96  15 | 15410  270  217  861  233  229  121  154  96  94  6  299  18  510  461  96  24  104  237  24  11  47  214  269  86  27 | 17582  531  166  2458  260  303  126  213  206  135  4  220  83  977  574  127  23  211  469  37  37  69  490  396  131  31 | 24654  1185  510  256  602  357  471  692  105  254  20  355  26  1183  794  432  36  503  353  87  49  120  323  306  168  38 | 18572  1963  977  529  644  555  898  1255  184  437  32  411  24  1193  869  540  93  522  470  34  46  161  326  276  187  81 | 20285  1588  492  345  584  653  1154  1403  117  289  24  452  38  1125  732  807  193  552  327  17  44  238  359  194  282  123 | 9151  4052  199  176  283  966  1370  1727  60  77  19  201  3  412  186  559  20  737  147  12  362  113  228  93  182  161 | 5952  2257  412  292  547  2404  1311  1655  104  197  19  145  15  337  196  276  58  225  88  47  28  92  102  53  208  300 | 5064  959  397  371  788  1242  1959  2553  84  334  6  214  13  589  289  651  14  150  167  17  22  125  94  60  223  275 | 6429  3600  946  871  795  1007  1099  1134  824  490  42  312  47  686  453  306  83  525  523  162  54  120  269  172  176  185 | 6620  3172  752  591  538  966  786  852  318  337  32  240  58  562  413  267  63  554  288  169  33  93  254  131  215  206 | 6965  4182  1227  1172  1351  1164  1203  1253  572  612  36  273  108  670  574  400  74  606  332  41  187  226  282  133  210  298 | 9504  2735  452  248  2065  2722  542  583  290  279  43  204  51  516  587  151  80  398  211  25  210  92  163  108  339  286 | 14534  2474  413  348  1058  3135  336  460  614  230  8  257  38  732  547  183  33  629  409  35  43  111  377  208  506  258 | 10020  1528  339  224  2417  2056  551  738  113  311  1  122  26  677  714  238  38  288  154  27  63  66  133  143  271  197 |

(B)

| OTUs No. | Description | Sequences of each sample which were represented by the OTUs | | | | | | | | | | | | | | | | | |
| --- | --- | --- | --- | --- | --- | --- | --- | --- | --- | --- | --- | --- | --- | --- | --- | --- | --- | --- | --- |
|  |  | SGK321_L21 | SGK321_L22 | SGK321_L23 | SGK321_T21 | SGK321_T22 | SGK321_T23 | SY321_L21 | SY321_L22 | SY321_L23 | SY321_T21 | SY321_T22 | SY321_T23 | XLZ13_L21 | XLZ13_L22 | XLZ13_L23 | XLZ13_T21 | XLZ13_T22 | XLZ13_T23 |
| 98  76  304  86  91  241  152  251  153  298  286  124  302  1  300  186  103  14  200  203  128  303  64  227  242  190 | *unclassified_o__Pezizales norank_o__Sordariales*  *Fusarium*  *norank_o__Hypocreales*  *Scopulariopsis*  *norank_p__Zygomycota*  *unclassified_p__Ascomycota*  *norank_f__Ascobolaceae*  *norank_c__Sordariomycetes*  *unclassified_f__Nectriaceae*  *unclassified_c__Agaricomycetes*  *unclassified_c__Dothideomycetes*  *Cryptococcus*  *unclassified_o__Pezizales*  *unclassified_f__Nectriaceae*  *unclassified_f__Trichocomaceae*  *Talaromyces*  *norank_o__Sordariales*  *unclassified_p__Ascomycota*  *unclassified_f__Auriculariaceae norank_c__Agaricomycetes*  *Mycosphaerella*  *unclassified_p__Ascomycota*  *unclassified_o__Pezizales*  *unclassified_d__Eukaryota*  *unclassified_k__Fungi* | 7036  5811  1559  1276  711  1311  1659  1382  744  607  423  774  297  453  558  816  651  1008  278  457  394  358  419  141  224  365 | 4776  4376  1368  2532  532  1294  1772  1435  1080  610  430  482  204  637  435  682  954  742  539  424  462  290  271  239  205  370 | 2824  4137  1272  1148  439  839  1440  1361  549  476  310  286  167  384  292  411  381  571  199  193  318  164  185  109  112  224 | 12442  2175  631  841  345  1417  845  810  379  334  1597  580  196  571  574  442  576  587  315  843  409  135  394  359  364  174 | 5037  1614  623  864  399  1569  1129  856  172  289  767  378  99  388  348  1379  1338  497  177  684  775  98  207  215  238  238 | 7732  3261  830  1268  637  2155  1443  1222  281  482  772  1112  90  594  609  624  547  722  322  478  374  207  421  240  394  418 | 2842  7258  1504  1326  503  1388  3348  3431  643  532  608  1445  107  123  118  511  1309  517  62  345  503  142  67  47  90  161 | 5228  6183  1566  1725  561  1334  1832  1746  556  632  818  408  98  376  330  638  992  703  203  761  1075  163  205  169  153  185 | 4511  5079  913  710  283  549  1848  1717  253  364  613  286  67  568  243  674  687  778  292  457  217  139  215  186  122  179 | 4300  6189  1596  1808  520  630  1613  1509  687  587  1039  1078  46  427  437  822  829  984  303  616  326  168  212  175  117  166 | 3213  3488  722  1002  295  473  742  756  338  299  620  845  11  258  228  417  484  516  172  539  158  79  165  134  64  108 | 3182  3684  1174  984  555  361  1562  1420  215  517  319  468  21  306  302  540  318  465  136  352  243  102  160  78  66  121 | 7539  6047  1410  1563  830  1295  1974  1699  785  639  359  777  181  698  536  679  535  834  423  262  427  429  340  171  242  298 | 5070  7185  1291  1454  640  2234  1238  1138  359  451  1529  569  121  417  359  445  415  858  182  302  774  277  262  185  231  367 | 2998  7500  1794  1454  446  1694  1775  1605  631  641  509  537  161  221  251  482  599  607  138  251  291  281  176  63  144  353 | 7890  5764  1091  1816  574  1012  368  378  398  413  556  1109  99  652  558  178  292  1024  344  380  417  161  483  214  247  176 | 2005  3865  2850  5316  320  738  641  414  612  786  755  842  642  202  353  1335  1242  770  196  201  179  147  205  156  110  238 | 6954  5479  1115  1867  536  1084  904  1141  735  395  992  800  151  249  135  263  692  282  105  512  219  167  83  108  47  33 |

(C)

| OTUs No. | Description | Sequences of each sample which were represented by the OTUs | | | | | | | | | | | | | | | | | |
| --- | --- | --- | --- | --- | --- | --- | --- | --- | --- | --- | --- | --- | --- | --- | --- | --- | --- | --- | --- |
|  |  | SGK321_L31 | SGK321_L32 | SGK321_L33 | SGK321_T31 | SGK321_T32 | SGK321_T33 | SY321_L31 | SY321_L32 | SY321_L33 | SY321_T31 | SY321_T32 | SY321_T33 | XLZ13_L31 | XLZ13_L32 | XLZ13_L33 | XLZ13_T31 | XLZ13_T32 | XLZ13_T33 |
| 98  76  304  86  91  241  152  251  153  298  286  124  302  1  300  186  103  14  200  203  128  303  64  227  242  190 | *unclassified_o__Pezizales norank_o__Sordariales*  *Fusarium*  *norank_o__Hypocreales*  *Scopulariopsis*  *norank_p__Zygomycota*  *unclassified_p__Ascomycota*  *norank_f__Ascobolaceae*  *norank_c__Sordariomycetes*  *unclassified_f__Nectriaceae*  *unclassified_c__Agaricomycetes*  *unclassified_c__Dothideomycetes*  *Cryptococcus*  *unclassified_o__Pezizales*  *unclassified_f__Nectriaceae*  *unclassified_f__Trichocomaceae*  *Talaromyces*  *norank_o__Sordariales*  *unclassified_p__Ascomycota*  *unclassified_f__Auriculariaceae norank_c__Agaricomycetes*  *Mycosphaerella*  *unclassified_p__Ascomycota*  *unclassified_o__Pezizales*  *unclassified_d__Eukaryota*  *unclassified_k__Fungi* | 5315  2893  665  744  253  802  398  465  402  234  503  424  307  227  248  150  540  418  153  1105  483  258  168  153  86  85 | 3837  3507  944  779  340  1494  1480  1763  400  319  722  539  454  246  198  472  826  487  187  543  523  266  133  108  154  236 | 7553  4026  715  678  377  1507  534  681  339  259  735  702  425  567  277  263  602  696  334  493  315  206  268  224  225  168 | 4323  2489  589  937  247  1838  1040  1082  191  276  180  542  85  427  195  265  156  336  207  371  394  150  212  137  282  212 | 6105  4876  941  905  732  3360  1208  1347  235  399  1213  579  125  517  315  274  137  561  248  271  473  138  230  174  396  350 | 11748  2214  858  742  400  2361  904  1097  232  335  400  974  215  682  458  381  168  454  261  449  220  144  239  265  400  216 | 8119  3594  1175  3334  356  1632  639  794  465  373  1909  509  539  555  511  364  437  760  343  338  805  421  366  344  270  178 | 11414  2582  1053  1018  258  1383  1140  1276  985  392  406  563  425  949  419  520  311  557  642  870  479  492  409  391  277  180 | 5009  1937  719  950  177  1199  642  593  1005  249  401  449  326  384  242  261  283  339  403  206  364  291  235  202  194  167 | 7868  3498  857  594  353  1959  927  1040  471  295  716  672  429  543  326  358  259  566  322  282  233  291  248  195  316  217 | 3237  4350  1185  728  443  2286  1856  2085  331  381  1026  862  276  344  191  364  386  464  174  641  1978  238  113  155  215  313 | 4842  3021  1015  882  580  1887  929  985  443  387  1356  1627  327  355  309  327  453  483  207  1296  148  293  143  201  262  208 | 4244  3591  1735  1215  596  2264  882  851  964  552  4396  509  1687  219  285  310  192  372  190  233  296  715  221  193  244  299 | 3957  2685  1572  1205  499  1842  776  901  790  468  4647  413  826  262  274  222  181  311  233  413  372  282  114  213  182  185 | 3469  2968  1517  1060  433  1627  768  812  775  396  2769  369  1645  259  269  207  194  300  214  210  289  466  143  132  162  223 | 5327  1757  858  775  391  1408  593  665  309  247  3365  708  1214  268  242  296  190  284  128  395  393  121  156  153  167  98 | 1030  1644  1036  779  710  1857  887  915  649  342  4648  955  1099  85  159  210  434  189  96  550  255  88  48  105  139  179 | 965  1321  1135  1025  748  1374  523  505  380  384  3846  1167  813  69  162  188  304  165  72  153  299  68  38  90  92  114 |

(D)

| OTUs No. | Description | Sequences of each sample which were represented by the OTUs | | | | | | | | | | | | | | | | | |
| --- | --- | --- | --- | --- | --- | --- | --- | --- | --- | --- | --- | --- | --- | --- | --- | --- | --- | --- | --- |
|  |  | SGK321_L41 | SGK321_L42 | SGK321_L43 | SGK321_T41 | SGK321_T42 | SGK321_T43 | SY321_L41 | SY321_L42 | SY321_L43 | SY321_T41 | SY321_T42 | SY321_T43 | XLZ13_L41 | XLZ13_L42 | XLZ13_L43 | XLZ13_T41 | XLZ13_T42 | XLZ13_T43 |
| 98  76  304  86  91  241  152  251  153  298  286  124  302  1  300  186  103  14  200  203  128  303  64  227  242  190 | *unclassified_o__Pezizales norank_o__Sordariales*  *Fusarium*  *norank_o__Hypocreales*  *Scopulariopsis*  *norank_p__Zygomycota*  *unclassified_p__Ascomycota*  *norank_f__Ascobolaceae*  *norank_c__Sordariomycetes*  *unclassified_f__Nectriaceae*  *unclassified_c__Agaricomycetes*  *unclassified_c__Dothideomycetes*  *Cryptococcus*  *unclassified_o__Pezizales*  *unclassified_f__Nectriaceae*  *unclassified_f__Trichocomaceae*  *Talaromyces*  *norank_o__Sordariales*  *unclassified_p__Ascomycota*  *unclassified_f__Auriculariaceae norank_c__Agaricomycetes*  *Mycosphaerella*  *unclassified_p__Ascomycota*  *unclassified_o__Pezizales*  *unclassified_d__Eukaryota*  *unclassified_k__Fungi* | 1245  678  7456  2769  1374  498  403  150  650  1551  13  763  897  144  315  413  926  136  102  25  17  362  41  48  21  78 | 969  566  7583  3199  836  408  569  247  571  1817  36  517  861  113  390  359  703  102  89  15  112  485  19  37  20  64 | 1758  1045  5930  4330  466  785  391  155  668  1720  10  769  529  162  575  384  494  167  123  50  24  799  95  40  65  160 | 544  290  6416  1294  41  4  441  99  1841  1092  50  408  1442  68  218  411  61  26  264  1  177  256  43  40  0  3 | 2114  1008  5891  2536  213  51  411  168  2026  1264  11  550  2837  206  562  562  594  219  427  8  8  360  130  55  7  26 | 1478  456  10340  3258  171  40  321  80  2764  1986  13  528  1999  176  524  612  106  49  403  2  8  269  88  52  3  16 | 1135  544  7080  2763  6874  42  342  145  1014  2226  6  765  652  298  569  502  788  162  127  6  4  328  25  32  5  12 | 1235  1103  8854  3324  353  93  363  113  722  1935  6  456  630  197  446  720  1347  280  188  11  12  490  34  59  4  26 | 943  531  7177  2880  2636  108  352  140  663  2084  10  544  680  196  471  387  719  131  114  6  12  331  25  35  9  25 | 700  399  7746  3107  232  54  320  105  4475  1333  83  1813  1927  56  284  798  1725  225  407  27  36  242  43  21  3  9 | 814  300  8307  3351  1627  45  565  198  1141  1339  93  978  1321  89  330  640  783  94  124  10  44  294  33  44  3  9 | 265  832  8174  4124  176  19  408  113  1265  1366  30  370  2682  20  148  571  677  92  150  3  13  457  26  7  3  11 | 4999  773  7377  3617  412  82  532  186  1691  1808  10  654  348  835  1188  909  456  207  1028  8  30  239  287  283  15  23 | 7725  950  7167  3570  291  60  540  221  1716  1642  20  838  705  964  1035  842  667  208  831  19  10  629  323  314  22  22 | 8320  609  6566  3067  206  54  557  230  1620  1490  10  737  411  1181  1347  958  464  183  1230  4  12  292  471  389  19  14 | 5093  678  6987  984  6766  48  554  174  539  1595  4  311  1682  1024  1136  714  146  101  368  24  117  204  121  198  7  18 | 3557  623  4320  2408  275  57  379  186  1824  865  4  797  2768  363  604  719  803  214  587  20  9  244  181  142  10  21 | 3241  444  3634  4161  958  36  194  60  704  761  1  315  1509  329  724  628  172  103  211  23  30  125  153  105  4  7 |

**Table S4.** Types and quantities of the secreted organic acids in the culture solution (μg/d) (*n* = 3).

| Organic acids | Hanhui 3T | Seedling  Hanhui 3 | Zhonghua11 | Hanhui 3T | Budding  Hanhui 3 | Zhonghua11 |
| --- | --- | --- | --- | --- | --- | --- |
| Formic acid  Malic acid  Acetic acid  Oxalic acid  **Citric acid** | 268±34  213±31  94±22  153±32  794±134 | 307±47  332±45  157±20  528±39  1787±332 | 230±37  395±43  116±26  748±69  1987±232 | 153±40  82±21  50±12  65±16  179±40 | 486±48  114±31  256±32  109±12  1145±234 | 321±34  123±20  94±32  253±12  894±134 |

^a^ Values represent the averages of three repetitions and the standard deviations.

Figure S1 Rarefaction curves of all of the rhizosphere samples based on OTUs


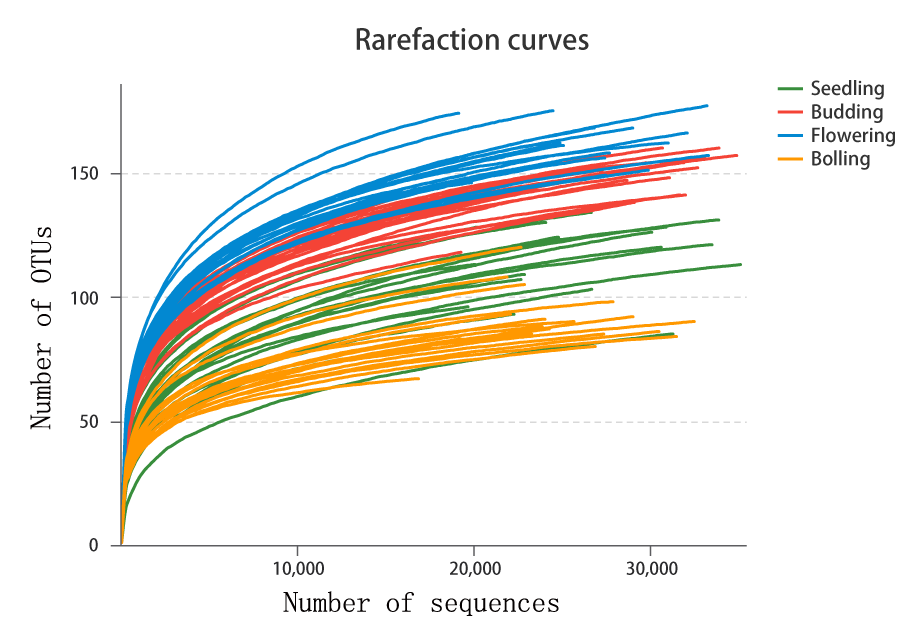


Figure S2 Analysis of similarities (ANOSIM) for fungal communities of the rhizosphere samples based on OTUs


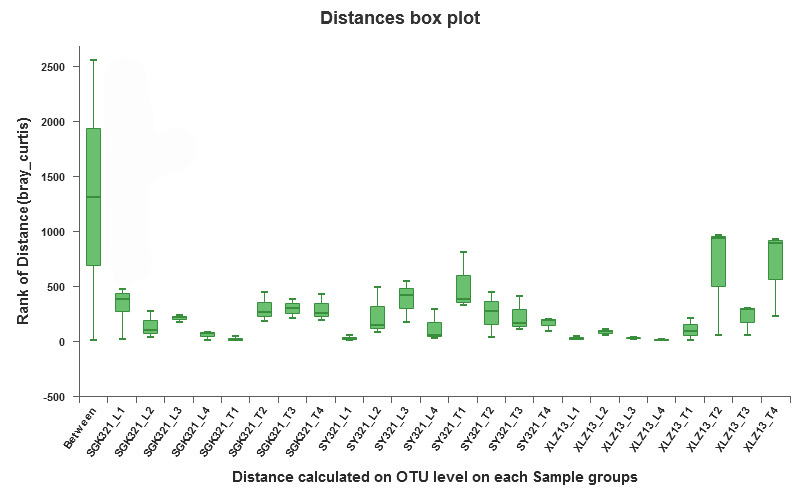


Figure S3 Hierarchical cluster analysis of the fungal communities from the rhizosphere of different cotton varieties and root environments at seedling (A), budding (B), flowering (C), and bolling (D) stages based on the dominant fungal genera, based on the Hellinger distances of microbial communities.


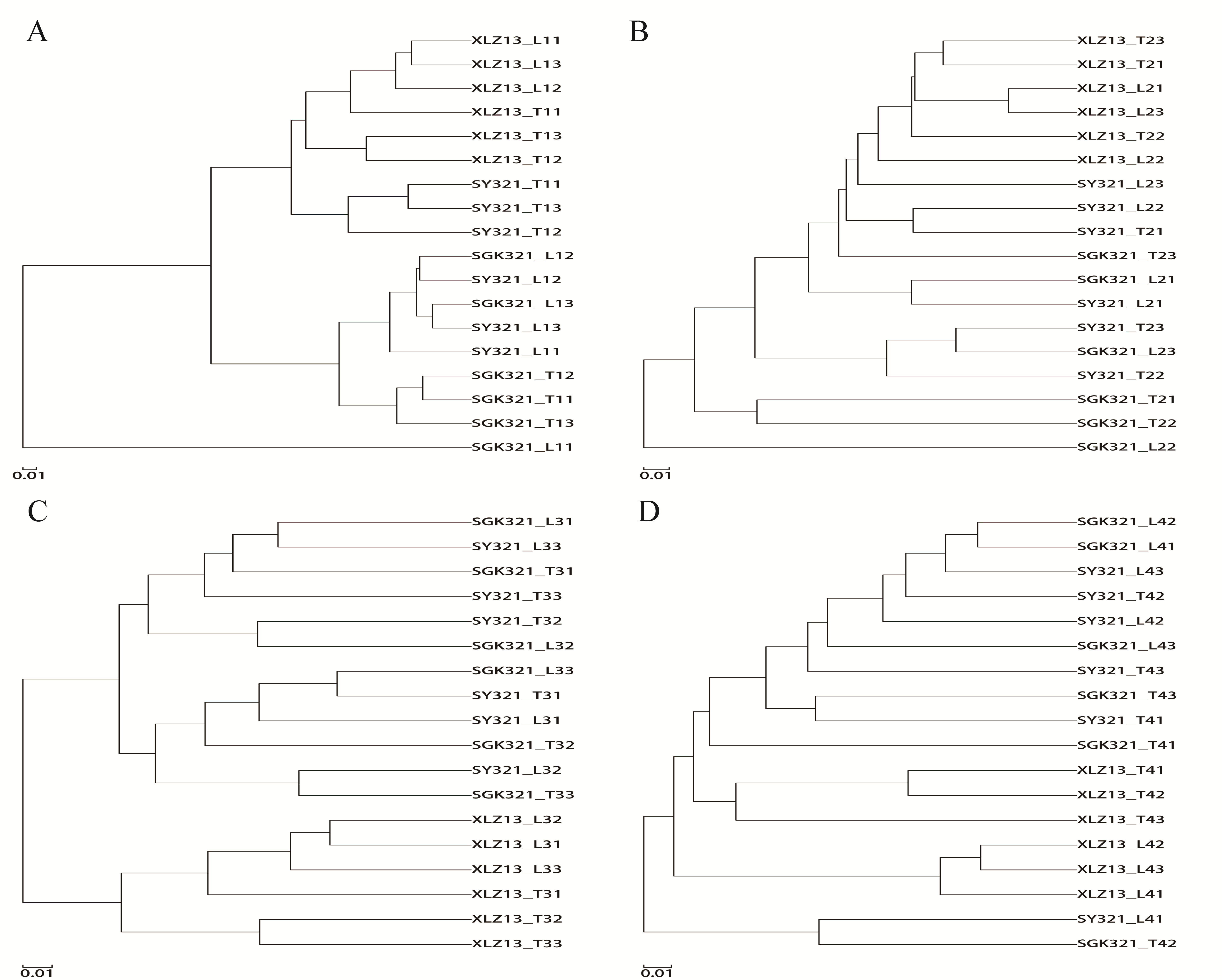


Figure S4 Hierarchical cluster analysis of the fungal communities from the rhizosphere of different cotton varieties and root environments at seedling (A), budding (B), flowering (C), and bolling (D) stages based on the rare fungal genera, based on the Hellinger distances of microbial communities.


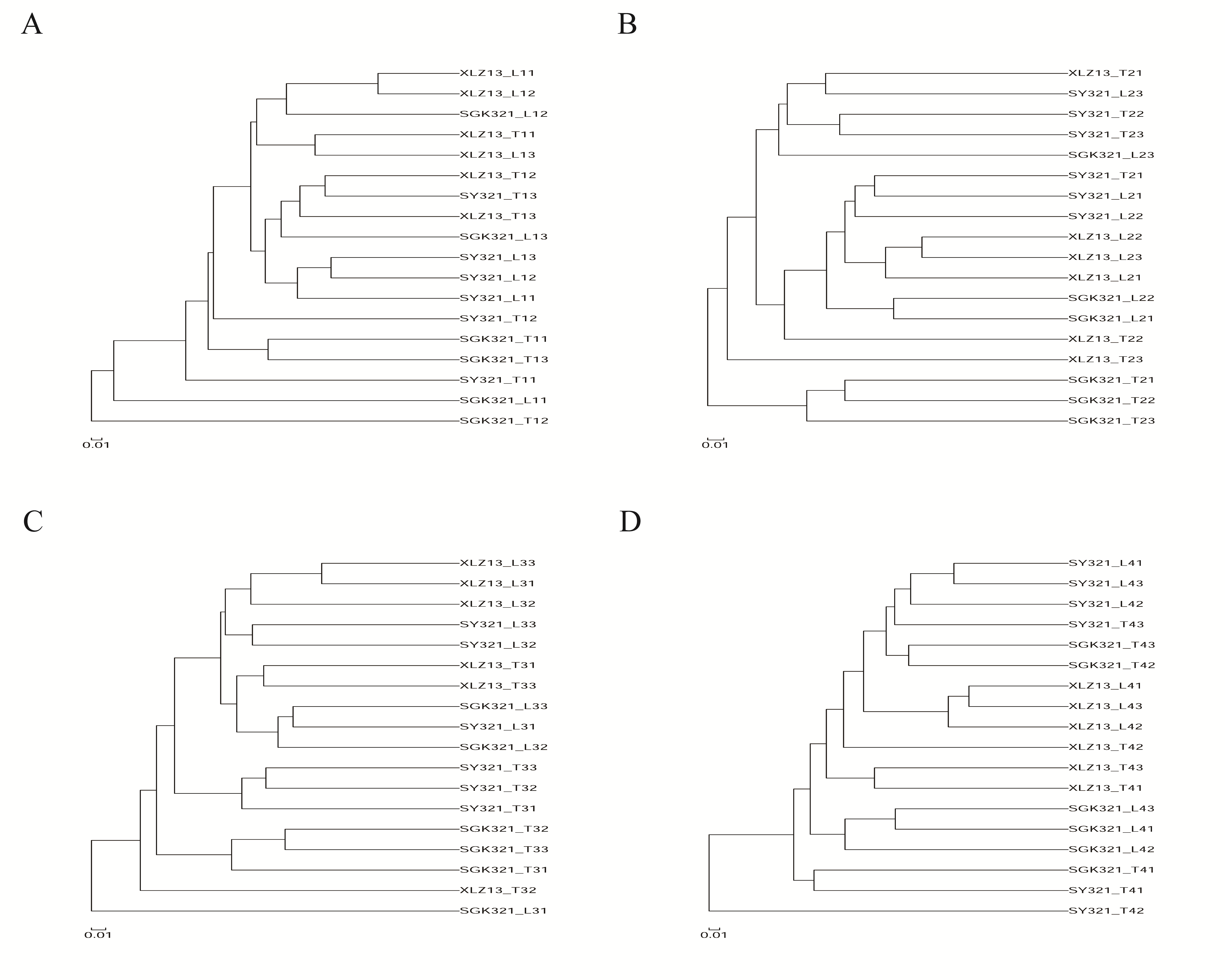


Figure S5 Hierarchical cluster analysis of the fungal communities from the rhizosphere of different cotton varieties and root environments at seedling (A), budding (B), flowering (C), and bolling (D) stages based on the responsive fungal genera, based on the Hellinger distances of microbial communities.


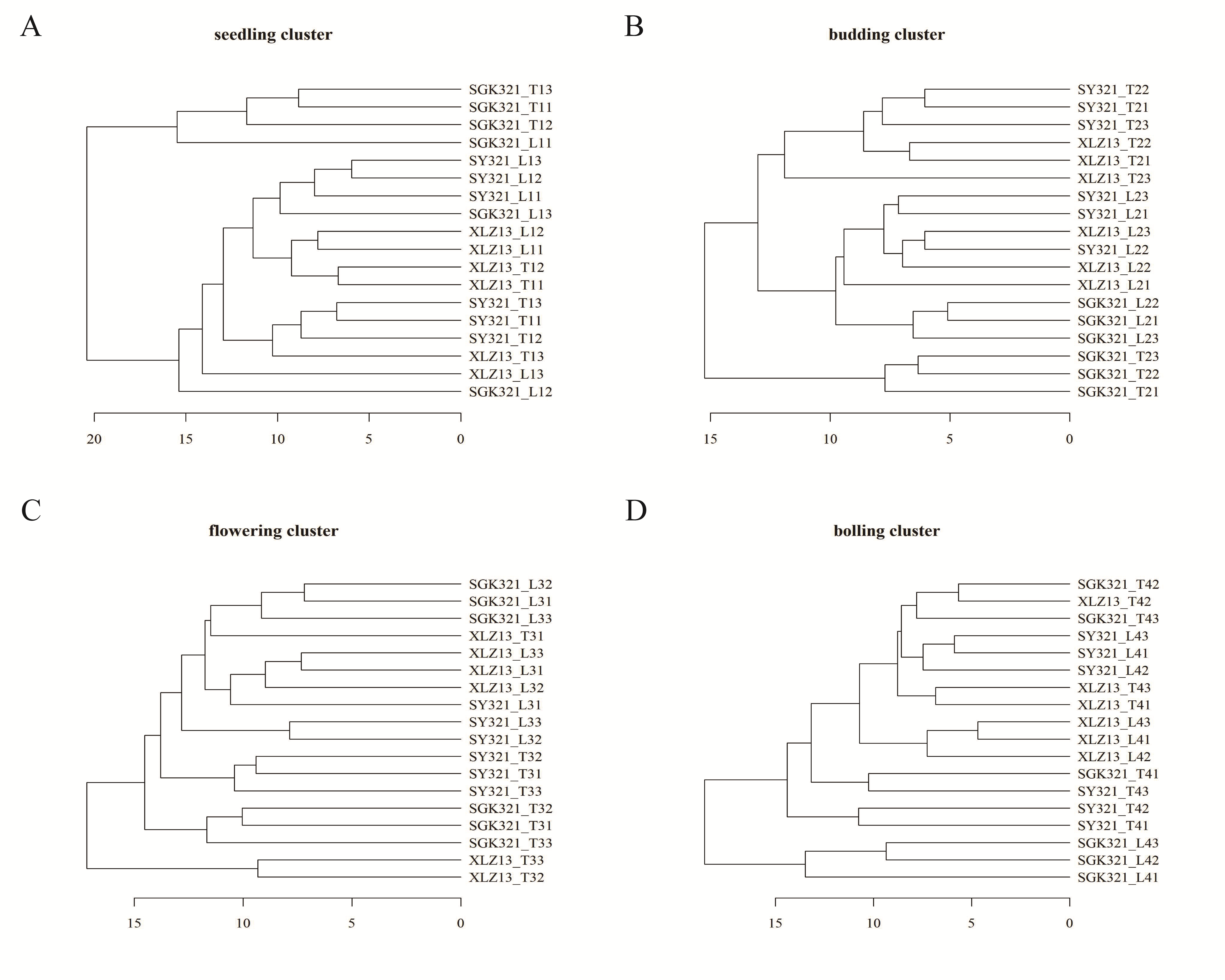


Figure S6 PCA analysis of fungal community at seedling (A), budding (B), flowering (C), and bolling (D) stages based on OTU level. BS indicates bulk soil control.


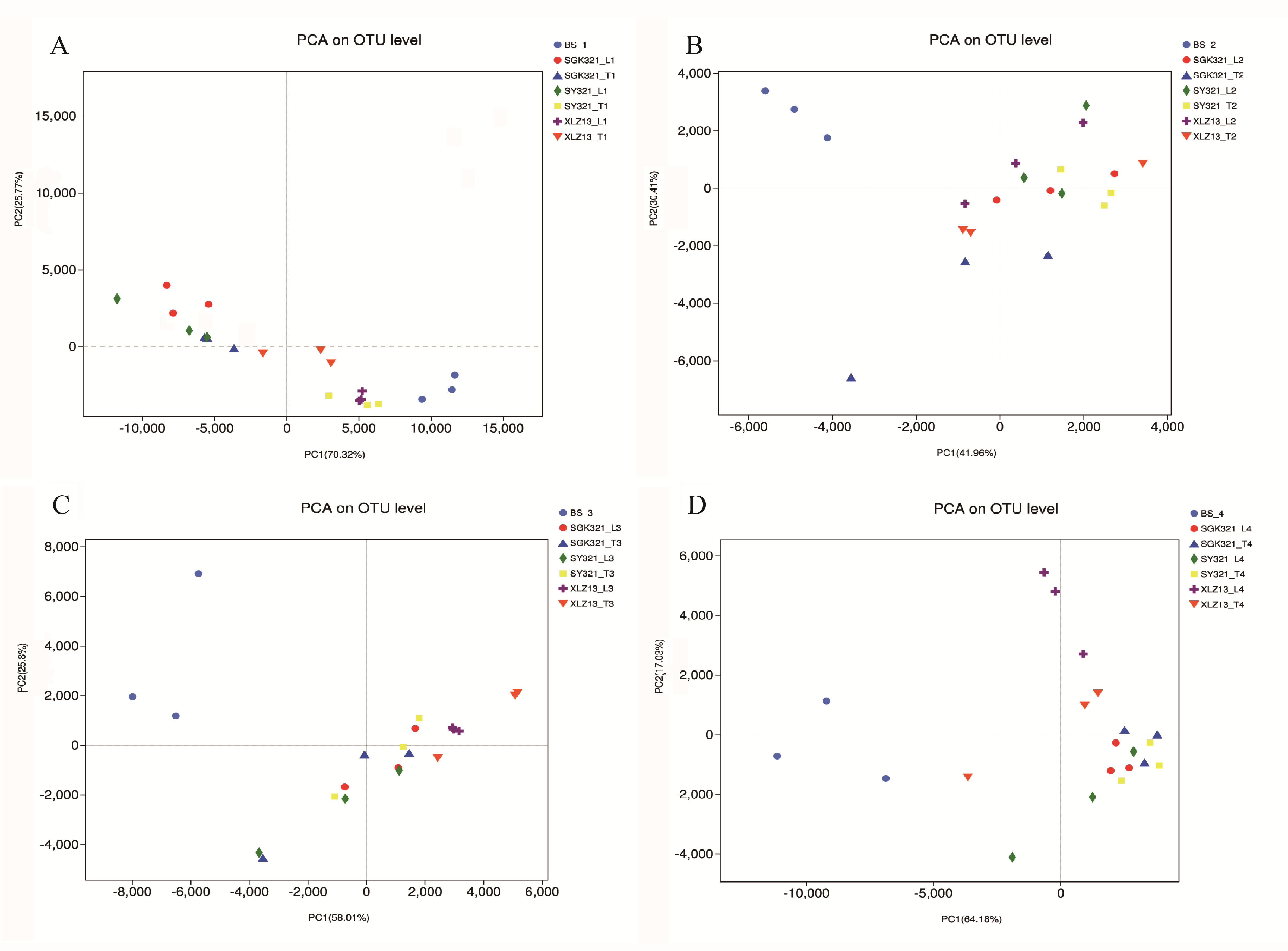


Figure S7 Bt protein contents of the different root tissues collected at different growth stages of the cotton varieties.

**
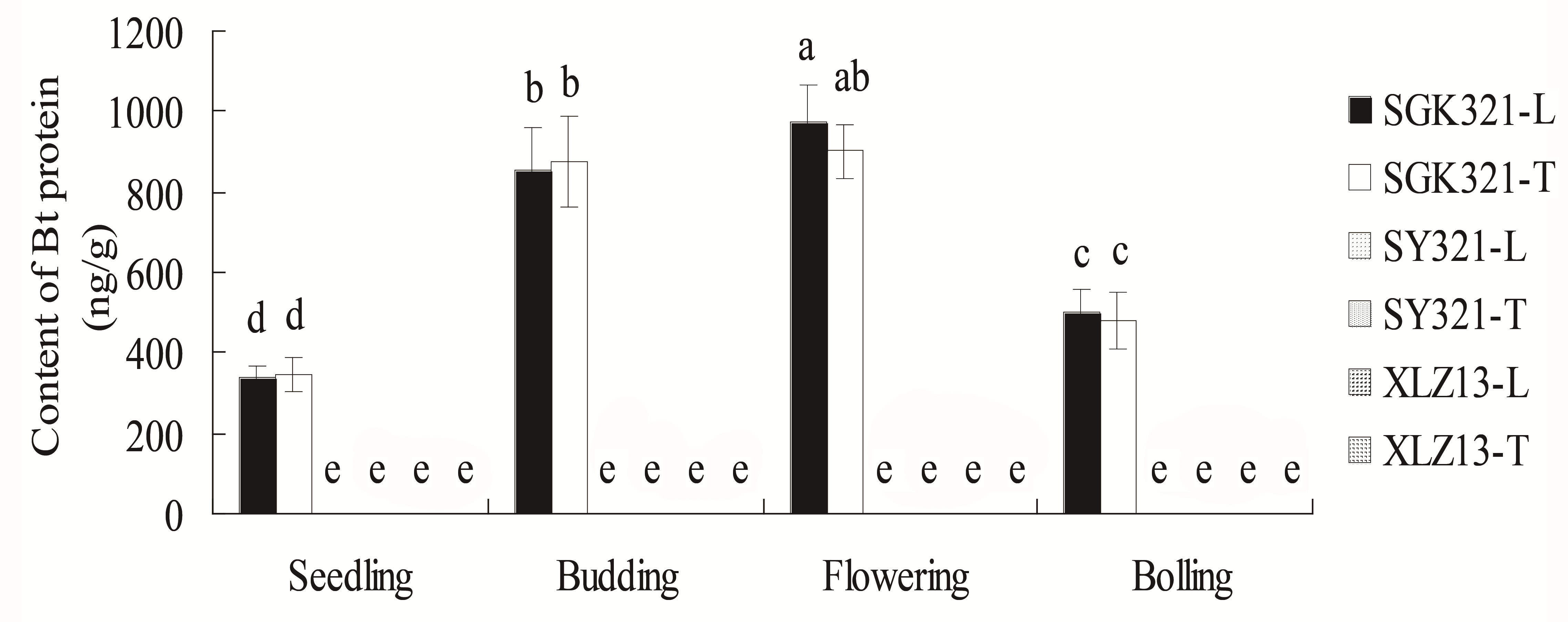
**
